# Optimum Achievable Rates in Two Random Number Generation Problems with *f*-Divergences Using Smooth Rényi Entropy †

**DOI:** 10.3390/e26090766

**Published:** 2024-09-06

**Authors:** Ryo Nomura, Hideki Yagi

**Affiliations:** 1 Center for Data Science, Waseda University, Tokyo 169-8050, Japan; 2 Department of Computer and Network Engineering, The University of Electro-Communications, Tokyo 182-8585, Japan; h.yagi@uec.ac.jp

**Keywords:** *f*-divergence, Hellinger distance, intrinsic randomness, Kullback–Leibler divergence, random number generation, smooth Rényi entropy, source resolvability, variational distance

## Abstract

Two typical fixed-length random number generation problems in information theory are considered for *general* sources. One is the source resolvability problem and the other is the intrinsic randomness problem. In each of these problems, the optimum achievable rate with respect to the given approximation measure is one of our main concerns and has been characterized using two different information quantities: the information spectrum and the smooth Rényi entropy. Recently, optimum achievable rates with respect to *f*-divergences have been characterized using the information spectrum quantity. The *f*-divergence is a general non-negative measure between two probability distributions on the basis of a convex function *f*. The class of *f*-divergences includes several important measures such as the variational distance, the KL divergence, the Hellinger distance and so on. Hence, it is meaningful to consider the random number generation problems with respect to *f*-divergences. However, optimum achievable rates with respect to *f*-divergences using the smooth Rényi entropy have not been clarified yet in both problems. In this paper, we try to analyze the optimum achievable rates using the smooth Rényi entropy and to extend the class of *f*-divergence. To do so, we first derive general formulas of the *first-order* optimum achievable rates with respect to *f*-divergences in both problems under the same conditions as imposed by previous studies. Next, we relax the conditions on *f*-divergence and generalize the obtained general formulas. Then, we particularize our general formulas to several specified functions *f*. As a result, we reveal that it is easy to derive optimum achievable rates for several important measures from our general formulas. Furthermore, a kind of *duality* between the resolvability and the intrinsic randomness is revealed in terms of the smooth Rényi entropy. *Second-order* optimum achievable rates and optimistic achievable rates are also investigated.

## 1. Introduction

Two typical fixed-length random number generation problems in information theory are considered for *general* sources. One is the source resolvability problem (i.e., the resolvability problem), and the other is the intrinsic randomness problem. The problem setting of the *resolvability* problem is as follows. Given an arbitrary source $X={\left\{X^{n}\right\}}_{n=1}^{\infty }$ (the *target* random number), we approximate it by using a discrete random number that is uniformly distributed, which we call the *uniform random number*. Here, the size of the uniform random number is requested to be as small as possible. In this setting, a degree of approximation is measured by several criteria. Han and Verdú [1] and Steinberg and Verdú [2] have determined the *first-order* optimum achievable rates with respect to the variational distance and the normalized Kullback–Leibler (KL) divergence. Nomura [3] has studied the *first-order* optimum achievable rates with respect to the KL divergence. Recently, Nomura [4] has characterized the *first-order* optimum achievable rates with respect to *f*-divergences. The class of *f*-divergence considered in [4] includes the variational distance and the KL divergence. Hence, the result can be considered as a generalization of the results given in [1,3]. The *second-order* optimum achievable rates in the resolvability problem have also been studied with respect to several approximation measures [4,5]. It should be noted that the results mentioned above are based on the information spectrum quantity. On the other hand, Uyematsu [6] has characterized the *first-order* optimum achievable rate with respect to the variational distance using the smooth Rényi entropy.

The *intrinsic randomness* problem, which is also one of typical random number generation problems, has also been studied. The problem setting of the intrinsic randomness problem is as follows. By using a given arbitrary source $X={\left\{X^{n}\right\}}_{n=1}^{\infty }$ (the *coin* random number), we approximate a discrete *uniform* random number whose size is requested to be as large as possible. Also in the intrinsic randomness problem, optimum achievable rates with respect to various criteria have been considered. Vembu and Verdú [7] have considered the intrinsic randomness problem with respect to the variational distance as well as the normalized KL divergence and derived *general formulas* of the *first-order* optimum achievable rates (cf. Han [8]). Hayashi [9] has considered the *first-* and *second-order* optimum achievable rates with respect to the KL divergence. Recently, the *first-* and *second-order* optimum achievable rates with respect to *f*-divergences have been clarified in [4]. The results mentioned here are based on information spectrum quantities. On the other hand, Uyematsu and Kunimatsu [10] have characterized the *first-order* optimum achievable rates with respect to the variational distance using the smooth Rényi entropy.

Related works include works given by Liu, Cuff and Verdú [11], Yagi and Han [12], Kumagai and Hayashi [13,14], and Yu and Tan [15]. In [11], the *channel* resolvability problem with respect to the $E_{\gamma }$-divergence has been considered. They have applied their results to the case of the *source* resolvability problem. Yagi and Han [16] have determined the optimum *variable-length* resolvability rates with respect to the variational distance as well as the KL divergence. Kumagai and Hayashi [13,14] have determined the *first- and second-order* optimum achievable rates in the *random number conversion* problem. It should be noted that the random number conversion problem includes the resolvability and intrinsic randomness problems treated in this paper. In [13,14], an approximation measure related to the Hellinger distance has been used. Yu and Tan [15] have considered the *random number conversion* problem with respect to the Rényi divergence.

As we have mentioned above, in both problems of the resolvability and the intrinsic randomness, various approximation measures have been considered. Furthermore, general formulas of achievable rates have been characterized by using the information spectrum quantity and the smooth Rényi entropy. We here note that optimum achievable rates with respect to *f*-divergence using the smooth Rényi entropy have not been clarified yet. The smooth Rényi entropy is an information quantity that has a clear operational meaning and is easy to understand. Moreover, a class of *f*-divergences is a general distance measure, in which several important measures are included. In this paper, hence, we try to characterize the *first-* and *second-order* optimum achievable rates with respect to *f*-divergences using the smooth Rényi entropy. In addition, we also extend the class of *f*-divergence for which optimum achievable rates can be characterized. As a result, we find that two types of smooth Rényi entropies are useful to describe these optimum achievable rates for a wider class of *f*-divergence. Furthermore, a kind of *duality* between the resolvability and the intrinsic randomness is revealed in terms of the smooth Rényi entropy and *f*-divergences.

This paper is organized as follows. In Section 2, we describe the problem setting and give some definitions of the optimum *first*-order achievable rates. The class of *f*-divergences and the smooth Rényi entropy are also introduced. In Section 3 and Section 4, we show *general formulas* of the optimum *first-order* achievable rates in the resolvability problem and the intrinsic randomness problem, respectively. In Section 5, we derive the general formulas of these achievable rates for an extended class of *f*-divergence. In Section 6, we apply general formulas obtained in previous sections to some specified functions *f* and compute the optimum *first-order* achievable rates in each cases. In Section 7, we show *general* formulas of the optimum *second*-order achievable rates in two problems. In Section 8, optimum achievable rates in the optimistic sense are considered. Section 9 is devoted to the discussion concerning our results. Finally, we provide some concluding remarks on our results in Section 10.

## 2. Preliminaries

### 2.1. f-Divergences

The *f*-divergence between two probability distributions $P_{Z}$ and $P_{\bar{Z}}$ is defined as follows [17]. Let f(t) be a convex function defined for t>0 and f(1)=0.

**Definition 1**  (*f*-divergence [17]). *Let $P_{Z}$ and $P_{\bar{Z}}$ denote probability distributions over a finite or countably infinite set Z. The f-divergence between $P_{Z}$ and $P_{\bar{Z}}$ is defined by*

(1) $$D_{f}\left(Z|\right|\bar{Z}):=\sum_{z\in Z}P_{\bar{Z}}\left(z\right)f\left(\frac{P_{Z}\left(z\right)}{P_{\bar{Z}}\left(z\right)}\right),$$

*where we set $0f\left(\frac{0}{0}\right)=0$, $f\left(0\right)=\lim_{t↓0}f\left(t\right)$, $0f\left(\frac{a}{0}\right)=\lim_{t\to 0}tf\left(\frac{a}{t}\right)=a\lim_{u\to \infty }\frac{f(u)}{u}$.*

The *f*-divergence is a general approximation measure, which includes some important measures. We give some examples of *f*-divergences [17,18]:

- f(t)=tlogt: (Kullback–Leibler (KL) divergence)
  (2) $$\begin{array}{c} D_{f}\left(Z|\right|\bar{Z})=\sum_{z\in Z}P_{Z}\left(z\right)\log\frac{P_{Z}\left(z\right)}{P_{\bar{Z}}\left(z\right)}=:D(Z||\bar{Z}). \end{array}$$
- f(t)=−logt: (Reverse Kullback–Leibler divergence)
  (3) $$\begin{array}{c} D_{f}\left(Z|\right|\bar{Z})=\sum_{z\in Z}P_{\bar{Z}}\left(z\right)\log\frac{P_{\bar{Z}}\left(z\right)}{P_{Z}\left(z\right)}=D(\bar{Z}||Z). \end{array}$$
- $f\left(t\right)=1-\sqrt{t}$: (Hellinger distance)
  (4) $$\begin{array}{c} D_{f}\left(Z|\right|\bar{Z})=1-\sum_{z\in Z}\sqrt{P_{Z}\left(z\right)P_{\bar{Z}}\left(z\right)}. \end{array}$$
- $f\left(t\right)={\left(1-\sqrt{t}\right)}^{2}$: (Squared Hellinger distance)
  (5) $$\begin{array}{c} D_{f}\left(Z|\right|\bar{Z})=\sum_{z\in Z}{\left(\sqrt{P_{Z}\left(z\right)}-\sqrt{P_{\bar{Z}}\left(z\right)}\right)}^{2}. \end{array}$$
- f(t)=|t−1|: (Variational distance)
  (6) $$\begin{array}{c} D_{f}\left(Z|\right|\bar{Z})=\sum_{z\in Z}\left|P_{Z}\left(z\right)-P_{\bar{Z}}\left(z\right)\right|. \end{array}$$
- $f\left(t\right)={\left(1-t\right)}^{+}:=\max\left\{1-t,0\right\}$: (Half variational distance)
  (7) $$\begin{array}{ccc} D_{f}\left(Z|\right|\bar{Z}) & = & \frac{1}{2}\sum_{z\in Z}\left|P_{Z}\left(z\right)-P_{\bar{Z}}\left(z\right)\right|\\ & = & \sum_{z\in Z:P_{Z}\left(z\right)>P_{\bar{Z}}\left(z\right)}\left(P_{Z}\left(z\right)-P_{\bar{Z}}\left(z\right)\right). \end{array}$$
- $f\left(t\right)=\frac{t^{\alpha }-\alpha t-\left(1-\alpha \right)}{\alpha (\alpha -1)}$: α-divergence (0<α<1)
  (8) $$\begin{array}{ccc} D_{f}\left(Z|\right|\bar{Z}) & = & \frac{1}{\alpha (\alpha -1)}\left(1-\sum_{z\in Z}P_{Z}{\left(z\right)}^{\alpha }P_{\bar{Z}}{\left(z\right)}^{1-\alpha }\right). \end{array}$$
- $f\left(t\right)={\left(t-\gamma \right)}^{+}$ : ($E_{\gamma }$-divergence) For any given γ≥1,
  (9) $$\begin{array}{c} D_{f}\left(Z|\right|\bar{Z})=\sum_{z\in Z:P_{Z}\left(z\right)>\gamma P_{\bar{Z}}\left(z\right)}\left(P_{Z}\left(z\right)-\gamma P_{\bar{Z}}\left(z\right)\right)=:E_{\gamma }\left(Z|\right|\bar{Z}). \end{array}$$

The $E_{\gamma }$-divergence is a generalization of the half variational distance defined in (7), because γ≥1 is arbitrary.

**Remark 1.**  *It is known [4] that the $E_{\gamma }$-divergence can be expressed as an f-divergence using the function:*

(10) $$f\left(t\right)={\left(\gamma -t\right)}^{+}+1-\gamma .$$

The following key property holds for the *f*-divergence from Jensen’s inequality [17]:

(11) $$\begin{array}{ccc} \sum_{z\in Z^{\prime }}b\left(z\right)f\left(\frac{a(z)}{b(z)}\right) & \geq & \left(\sum_{z\in Z^{\prime }}b\left(z\right)\right)f\left(\frac{\sum_{z\in Z^{\prime }}a\left(z\right)}{\sum_{z\in Z^{\prime }}b\left(z\right)}\right). \end{array}$$

As we have mentioned above, the *f*-divergence is a general approximation measure, which includes several important measures. In this study, we first assume the following conditions on the function *f* that have also been imposed by previous studies [4].

**C1)**  The function f(t) is a decreasing function for t>0 with f(0)>0.
**C2)**  The function f(t) satisfies
  (12) $$\lim_{u\to \infty }\frac{f(u)}{u}=0.$$
**C3)**  For any pair of positive real numbers (a,b), it holds that
  (13) $$\lim_{n\to \infty }\frac{f\left(e^{-nb}\right)}{e^{na}}=0.$$

**Remark 2.**  *Notice here that functions f(t)=−logt, $f\left(t\right)=1-\sqrt{t}$, and $f\left(t\right)={\left(1-t\right)}^{+}$ satisfy the above conditions, while f(t)=tlogt does not satisfy conditions C1) and C2). Moreover, it is not difficult to check that (10) satisfies these conditions.*

**Remark 3.**  *For a decreasing function f(t), it always holds that $f\left(0\right)=\lim_{t↓0}f\left(t\right)\geq 0$ because f(1)=0. Then, the condition f(0)>0 in C1) excludes the case of f(t)=0 for all t≥0, in which f-divergence is identically zero.*

**Remark 4.**  *From the definition of the f-divergence, C2) means*

(14) $$0f\left(\frac{a}{0}\right)=0,$$

*for any a>0. In the derivation of our main theorems, we can use (14) instead of (12).*

**Remark 5.**  *We will show in Section 5 that condition C1) is automatically met for the function f satisfying condition C2) (cf. claim (i) of Lemma 1).*

### 2.2. Smooth Rényi Entropy

In what follows, we consider the case of $Z=X^{n}$, where X is a finite or countably infinite set and *n* is an integer. We consider the *general* source defined as an infinite sequence

$$X={\left\{X^{n}=\left(X_{1}^{\left(n\right)},X_{2}^{\left(n\right)},\cdots ,X_{n}^{\left(n\right)}\right)\right\}}_{n=1}^{\infty }$$

of *n*-dimensional random variables $X^{n}$, where each component random variable $X_{i}^{\left(n\right)}$ takes values in a countable set X. Let $P_{X}\left(\cdot \right)$ denote the probability distribution of the random variable *X*. In this paper, we assume the following condition on the source X:

(15) $$\underline{H}\left(X\right)<+\infty ,$$

where

(16) $$\begin{array}{ccc} \underline{H}\left(X\right) & = & \sup\left\{R\left|\lim_{n\to \infty }\mathrm{Pr}\left\{\frac{1}{n}\log\frac{1}{P_{X^{n}}\left(X^{n}\right)}\geq R\right\}=1\right.\right\} \end{array}$$

is called the spectral inf-entropy rate of the source X [8]. Here, Han [8] ([Theorem 1.7.2]) has shown that

(17) $$\begin{array}{c} \underline{H}\left(X\right)\leq \log\left|X\right| \end{array}$$

holds. Hence, the condition (15) holds for any source with a *finite alphabet*.

The random number $U_{M}$ which is uniformly distributed on {1,2,⋯,M} is defined by

(18) $$P_{U_{M}}\left(i\right)=\frac{1}{M},\;\;i\in U_{M}:=\left\{1,2,\cdots ,M\right\}.$$

We next introduce the smooth Rényi entropy of the source.

**Definition 2**  (Smooth Rényi entropy of order α [19]). *For given random variables $X^{n}$, the smooth Rényi entropy of order α given δ(0≤δ<1) is defined by*

(19) $$H_{\alpha }\left(\delta |X^{n}\right):=\frac{1}{1-\alpha }\underset{P_{{\bar{X}}^{n}}\in B^{\delta }\left(P_{X^{n}}\right)}{\inf}\log\left(\sum_{x\in X^{n}}P_{{\bar{X}}^{n}}{\left(x\right)}^{\alpha }\right),$$

*where*

(20) $$\begin{array}{ccc} B^{\delta }\left(P_{X^{n}}\right) & := & \left\{P_{{\bar{X}}^{n}}\in P^{n}\left|\frac{1}{2}\sum_{x\in X^{n}}\left|P_{X^{n}}\left(x\right)-P_{{\bar{X}}^{n}}\left(x\right)\right|\leq \delta \right.\right\}.\;\; \end{array}$$

Here, $H_{\alpha }\left(\delta |X^{n}\right)$ is a decreasing function of δ. The smooth Rényi entropy of order 0 and the smooth Rényi entropy of order *∞* are, respectively, called the smooth max entropy and the smooth min entropy [20].

The following theorems have shown alternative expressions of the smooth max entropy and the smooth min entropy.

**Theorem 1**  (Uyematsu [6,21]).

(21) $$H_{0}\left(\delta |X^{n}\right)=\min_{\begin{array}{c} A_{n}\subset X^{n}\\ \mathrm{Pr}\{X^{n}\in A_{n}\}\geq 1-\delta \end{array}}\log\left|A_{n}\right|.$$

**Theorem 2**  (Uyematsu and Kunimatsu [10]).

(22) $$H_{\infty }\left(\delta |X^{n}\right)=-\underset{\begin{array}{c} \beta \geq \frac{1}{|X^{n}|}:\\ \sum_{x\in X^{n}}{\left(P_{X^{n}}\left(x\right)-\beta \right)}^{+}\leq \delta \end{array}}{\inf}\log\beta ,$$

*where if |X| is a countably infinite set, the infimum is taken over β≥0.*

**Figure 1:**
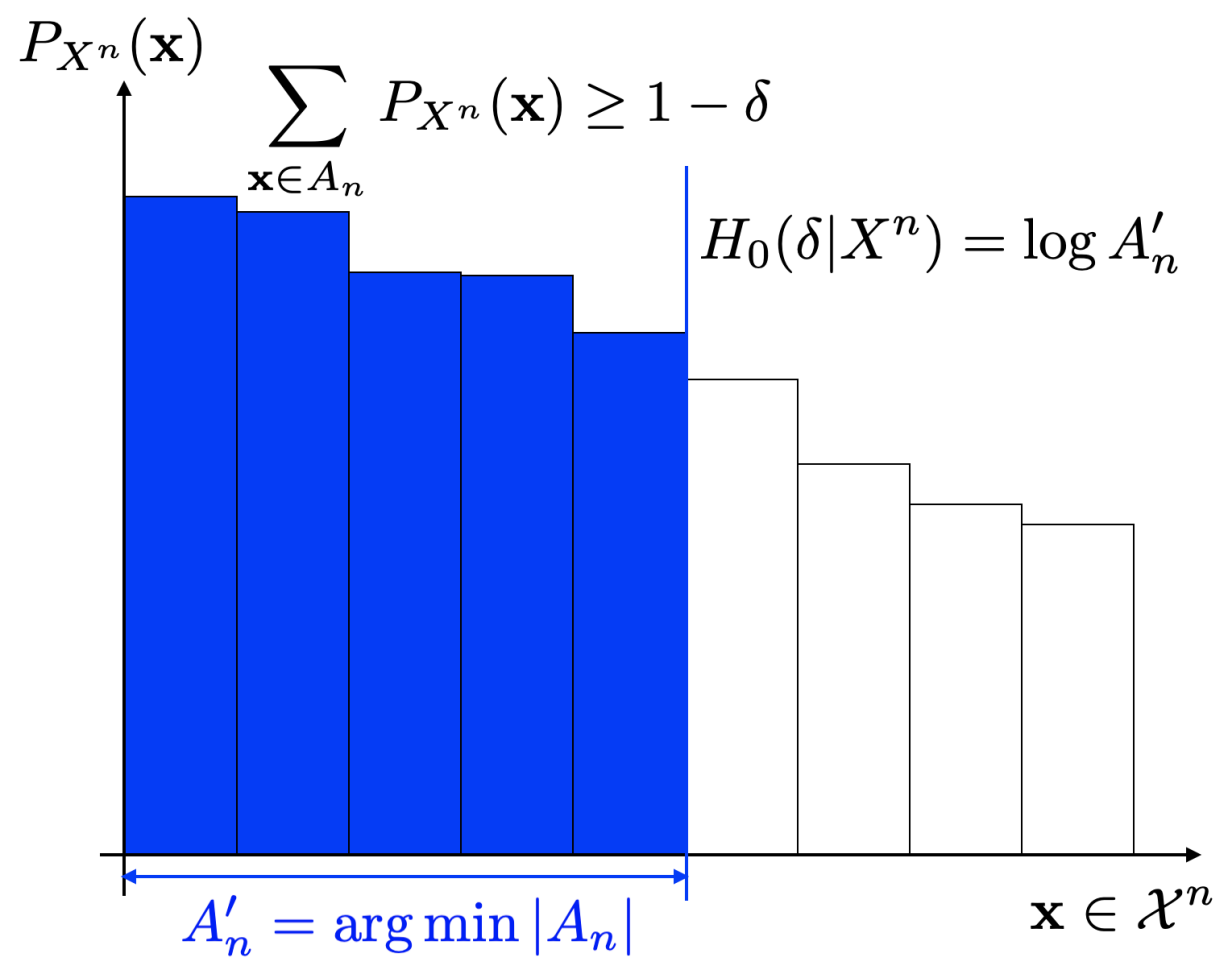
Smooth max entropy $H_{0}\left(\delta |X^{n}\right)$.

**Figure 2:**
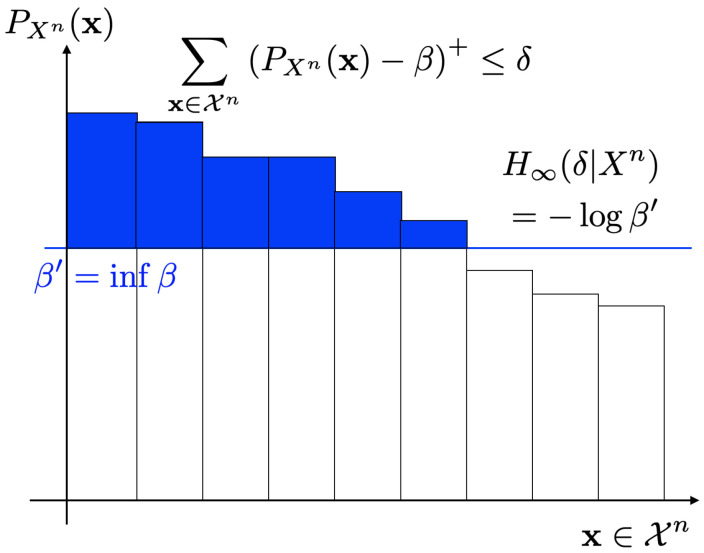
Smooth min entropy $H_{\infty }\left(\delta |X^{n}\right)$.

It should be noted that these alternative expressions are simple and easy to understand compared to (19). Figure 1 and Figure 2 show operational meanings of (21) and (22). As in Figure 1, the smooth max entropy $H_{0}\left(\delta |X^{n}\right)$ is equal to the logarithm of the cardinality of the set $A_{n}$ with $\mathrm{Pr}\{X^{n}\in A_{n}\}\geq 1-\delta$ where each of the sequence $x\in A_{n}$ has large probability. On the other hand, the smooth min entropy $H_{\infty }\left(\delta |X^{n}\right)$ is equal to the supremum of −logβ such that the sum of probabilities of sequences $x\in X^{n}$ that exceeds β is less than or equal to δ (Figure 2).

In this paper, we use the above alternative expressions of the smooth max entropy and the smooth min entropy instead of (19).

## 3. Source Resolvability Problem

**Figure 3:**
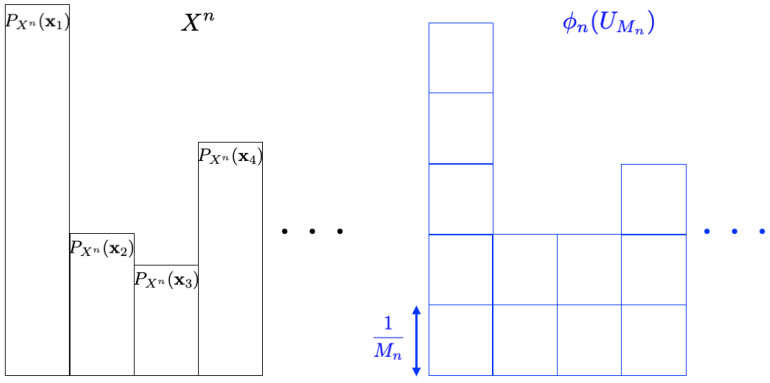
Resolvability problem.

We consider the problem concerning how to simulate a given discrete source $X={\left\{X^{n}\right\}}_{n=1}^{\infty }$ by using the uniform random number $U_{M_{n}}$ and the mapping $\phi_{n}$. Figure 3 is an illustrative figure of this problem (the probability distribution for $X^{n}$ is depicted in black, while the one for $\phi_{n}\left(U_{M_{n}}\right)$ is shown in blue). Since it is hard to simulate the exact source in general, we consider the approximation problem under some measure. This problem is called the resolvability problem. One of the main objectives in the resolvability problem is to derive the smallest value of *a* in the form of $M_{n}=e^{na}$, which we call the optimum resolvability rate [1,8]. This is formulated as follows.

**Definition 3.**  *Rate R is said to be D-achievable with the given f-divergence if there exists a sequence of mapping $\phi_{n}:U_{M_{n}}\to X^{n}$ such that*

(23) $$\underset{n\to \infty }{lim sup}D_{f}(X^{n}\left|\right|\phi_{n}\left(U_{M_{n}}\right))\leq D,$$

(24) $$\underset{n\to \infty }{lim sup}\frac{1}{n}\log M_{n}\leq R.$$

Given some *D*, if the rate constraint *R* is sufficiently large, it can be shown that there exists a sequence of mappings satisfying constraints in the above definition. Conversely, if *R* is too small, no sequence of mappings that satisfies constraints can be found. Therefore, in the resolvability problem, the infimum of *R* is of particular interest.

**Definition 4** (First-order optimum resolvability rate).
(25) $$\begin{array}{ccc} S_{r}^{\left(f\right)}\left(D|X\right) & := & \inf\left\{R\left|R\;is\;D-achievable\;with\;the\;given\;f-divergence\right.\right\}. \end{array}$$

**Remark 6.**  *It should be noted that we do not use $D_{f}(\phi_{n}\left(U_{M_{n}}\right)\left|\right|X^{n})$ but $D_{f}(X^{n}\left|\right|\phi_{n}\left(U_{M_{n}}\right))$ as a condition in Definition 3. This is important to consider the asymmetric measure such as the KL-divergence.*

**Remark 7.**  *We consider the case where D is in [0,f(0)) under the given f-divergence. Since f(t) is defined in the range t>0 and we assume that the function f(t) is a decreasing function of t, $D_{f}(X^{n}\left|\right|Y^{n})\leq f\left(0\right)$ holds for any distributions $P_{X^{n}}\left(\cdot \right)$ and $P_{Y^{n}}\left(\cdot \right)$ from the definition of f-divergence. Hence, D≥f(0) means that there exists no restriction about the approximation error (for example, f(0)=1 in the case of the half variational distance and f(0)=∞ in the case of the KL divergence). This case leads to the trivial result that the first-order optimum resolvability rate equals 0. Hence, we only consider the case of D∈[0,f(0)). A similar observation is applicable throughout the following sections.*

Our main objective in this section is to derive the general formula of the first-order optimum resolvability rate. To do so, we first derive the following two theorems. We use the notation $f^{-1}\left(a\right)=\inf\left\{t|f\left(t\right)=a\right\}.$

**Theorem 3.**  *Under conditions C1)–C3), for any γ>0 and any $M_{n}$ satisfying*

(26) $$\frac{1}{n}\log M_{n}\geq \frac{1}{n}H_{0}\left(1-f^{-1}\left(D\right)|X^{n}\right)+\gamma ,$$

*there exists a mapping $\phi_{n}$, which satisfies*

(27) $$D_{f}(X^{n}\left|\right|\phi_{n}\left(U_{M_{n}}\right))\leq D+\gamma$$

*for sufficiently large n.*

**Proof.**  We arbitrarily fix $M_{n}$ satisfying (26). We show that there exists a mapping $\phi_{n}$ that satisfies (27) for sufficiently large *n*. Let $B_{n}\subset X^{n}$ denote a set satisfying

(28) $$\mathrm{Pr}\left\{X^{n}\in B_{n}\right\}\geq f^{-1}\left(D\right)$$

and

(29) $$\log|B_{n}|=H_{0}\left(1-f^{-1}\left(D\right)|X^{n}\right).$$

The existence of the above set $B_{n}$ is guaranteed by (21). We define the probability distribution $P_{{\bar{X}}^{n}}$ over $B_{n}$ as

(30) $$P_{{\bar{X}}^{n}}\left(x\right):=\left\{\begin{array}{cc} \frac{P_{X^{n}}\left(x\right)}{\mathrm{Pr}\{X^{n}\in B_{n}\}} & x\in B_{n},\\ 0 & otherwise. \end{array}\right.$$

Furthermore, let a set $C_{n}$ be as

(31) $$C_{n}:=\left\{x\in B_{n}\left|P_{{\bar{X}}^{n}}\left(x\right)\geq \frac{1}{M_{n}}\right.\right\}$$

and arrange elements in $C_{n}$ as

(32) $$C_{n}=\left\{x_{1},x_{2},\cdots ,x_{|C_{n}|}\right\}$$

according to $P_{{\bar{X}}^{n}}\left(x\right)$ in ascendant order. That is, $P_{{\bar{X}}^{n}}\left(x_{i}\right)\leq P_{{\bar{X}}^{n}}\left(x_{j}\right)\;(1\leq i<j\leq |C_{n}\left|\right)$ holds. Here, we define $i^{*}:=\left|C_{n}\right|$ and index $x\in B_{n}\setminus C_{n}$ as $x_{i^{*}+1},x_{i^{*}+2},\cdots ,x_{|B_{n}|}$ arbitrarily.

Then, from the above definition, it holds that

(33) $$P_{{\bar{X}}^{n}}\left(x_{i^{*}}\right)=\max_{x\in C_{n}}P_{{\bar{X}}^{n}}\left(x\right).$$

Thus, from the assumption (15), for any small $\varepsilon \in (0,\underline{H}\left(X\right))$, it holds that

(34) $$P_{{\bar{X}}^{n}}\left(x_{i^{*}}\right)\geq e^{-n(\underline{H}\left(X\right)-\varepsilon )}$$

for sufficiently large *n*.

Set $k_{0}=0$. For $x_{1}$ we determine $k_{1}$ such that

(35) $$\frac{k_{1}}{M_{n}}\leq P_{{\bar{X}}^{n}}\left(x_{1}\right),\;\frac{k_{1}+1}{M_{n}}>P_{{\bar{X}}^{n}}\left(x_{1}\right).$$

Secondly, we determine $k_{2}$ for $x_{2}$ such that

(36) $$\frac{k_{2}-k_{1}}{M_{n}}\leq P_{{\bar{X}}^{n}}\left(x_{2}\right),\;\frac{k_{2}-k_{1}+1}{M_{n}}>P_{{\bar{X}}^{n}}\left(x_{2}\right).$$

In a similar way, we repeat this operation to choose $k_{i}$ for $x_{i}$ as long as possible. Then, it is not difficult to check that the above procedure does not stop before $i<i^{*}$.

We define a mapping $\phi_{n}:U_{M_{n}}\to X^{n}$ as

(37) $$\phi_{n}\left(j\right)=\left\{\begin{array}{cc} x_{i} & k_{i-1}+1\leq j\leq k_{i},\;i<i^{*}\\ x_{i^{*}} & \mathrm{otherwise} \end{array}\right.$$

and set ${\tilde{X}}^{n}=\phi_{n}\left(U_{M_{n}}\right)$.

We evaluate the performance of the mapping $\phi_{n}$. From the construction of the mapping, for any *i* satisfying $1\leq i\leq i^{*}-1$ it holds that

(38) $$P_{{\tilde{X}}^{n}}\left(x_{i}\right)\leq P_{{\bar{X}}^{n}}\left(x_{i}\right)$$

(39) $$P_{{\bar{X}}^{n}}\left(x_{i}\right)<P_{{\tilde{X}}^{n}}\left(x_{i}\right)+\frac{1}{M_{n}}.$$

We next evaluate $P_{{\tilde{X}}^{n}}\left(x_{i^{*}}\right)$. From the construction, we have $P_{{\bar{X}}^{n}}\left(x_{i^{*}}\right)\leq P_{{\tilde{X}}^{n}}\left(x_{i^{*}}\right)$. Since $P_{{\tilde{X}}^{n}}\left(x_{i}\right)=0$ holds for $\forall i\in B_{n}\setminus C_{n}$, we obtain

(40) $$P_{{\bar{X}}^{n}}\left(x_{i}\right)-P_{{\tilde{X}}^{n}}\left(x_{i}\right)=P_{{\bar{X}}^{n}}\left(x_{i}\right)<\frac{1}{M_{n}}$$

for $\forall i\in B_{n}\setminus C_{n}$. Hence, also from the construction of the mapping, we obtain

(41) $$\begin{array}{ccc} P_{{\tilde{X}}^{n}}\left(x_{i^{*}}\right)-P_{{\bar{X}}^{n}}\left(x_{i^{*}}\right) & = & \left(1-\sum_{i=1}^{i^{*}-1}P_{{\tilde{X}}^{n}}\left(x_{i}\right)\right)-\left(1-\sum_{x_{i}\in B_{n}\setminus \left\{x_{i^{*}}\right\}}P_{{\bar{X}}^{n}}\left(x_{i}\right)\right)\\ & = & \sum_{x_{i}\in B_{n}\setminus \left\{x_{i^{*}}\right\}}P_{{\bar{X}}^{n}}\left(x_{i}\right)-\sum_{x_{i}\in B_{n}\setminus \left\{x_{i^{*}}\right\}}P_{{\tilde{X}}^{n}}\left(x_{i}\right)\\ & = & \sum_{x_{i}\in B_{n}\setminus \left\{x_{i^{*}}\right\}}\left(P_{{\bar{X}}^{n}}\left(x_{i}\right)-P_{{\tilde{X}}^{n}}\left(x_{i}\right)\right)\\ & \leq & \frac{|B_{n}|}{M_{n}}\\ & \leq & e^{-n\gamma }, \end{array}$$

where the second equality is from the fact that $P_{{\tilde{X}}^{n}}\left(x_{i}\right)=0$ for $\forall i\in B_{n}\setminus C_{n}$, the first inequality is due to (39) and (40), and the last inequality is obtained from (26) and (29). Thus, we have

(42) $$P_{{\tilde{X}}^{n}}\left(x_{i^{*}}\right)\leq P_{{\bar{X}}^{n}}\left(x_{i^{*}}\right)+e^{-n\gamma }.$$

From the above argument, the *f*-divergence is given by

(43) $$\begin{array}{ccc} D_{f}\left(X^{n}\left|\right|\phi_{n}\left(U_{M_{n}}\right)\right) & = & \sum_{i=1}^{i^{*}}P_{{\tilde{X}}^{n}}\left(x_{i}\right)f\left(\frac{P_{X^{n}}\left(x_{i}\right)}{P_{{\tilde{X}}^{n}}\left(x_{i}\right)}\right)\\ & = & \sum_{i=1}^{i^{*}}P_{{\tilde{X}}^{n}}\left(x_{i}\right)f\left(\frac{P_{{\bar{X}}^{n}}\left(x_{i}\right)\mathrm{Pr}\left\{X^{n}\in B_{n}\right\}}{P_{{\tilde{X}}^{n}}\left(x_{i}\right)}\right)\\ & = & \sum_{i=1}^{i^{*}-1}P_{{\tilde{X}}^{n}}\left(x_{i}\right)f\left(\frac{P_{{\bar{X}}^{n}}\left(x_{i}\right)\mathrm{Pr}\left\{X^{n}\in B_{n}\right\}}{P_{{\tilde{X}}^{n}}\left(x_{i}\right)}\right)\\ & & +P_{{\tilde{X}}^{n}}\left(x_{i^{*}}\right)f\left(\frac{P_{{\bar{X}}^{n}}\left(x_{i^{*}}\right)\mathrm{Pr}\left\{X^{n}\in B_{n}\right\}}{P_{{\tilde{X}}^{n}}\left(x_{i^{*}}\right)}\right)\\ & \leq & \sum_{i=1}^{i^{*}-1}P_{{\tilde{X}}^{n}}\left(x_{i}\right)f\left(\mathrm{Pr}\left\{X^{n}\in B_{n}\right\}\right)\\ & & +P_{{\tilde{X}}^{n}}\left(x_{i^{*}}\right)f\left(\frac{P_{{\bar{X}}^{n}}\left(x_{i^{*}}\right)\mathrm{Pr}\left\{X^{n}\in B_{n}\right\}}{P_{{\tilde{X}}^{n}}\left(x_{i^{*}}\right)}\right), \end{array}$$

where the first equality is due to the condition C2) and the last inequality is due to (38) and the condition C1).

The second term of the RHS of (43) is evaluated as follows. From (42) and C1), we have

(44) $$\begin{array}{c} P_{{\tilde{X}}^{n}}\left(x_{i^{*}}\right)f\left(\frac{P_{{\bar{X}}^{n}}\left(x_{i^{*}}\right)\mathrm{Pr}\left\{X^{n}\in B_{n}\right\}}{P_{{\tilde{X}}^{n}}\left(x_{i^{*}}\right)}\right)\\ \;\leq \left(P_{{\bar{X}}^{n}}\left(x_{i^{*}}\right)+e^{-n\gamma }\right)f\left(\frac{P_{{\bar{X}}^{n}}\left(x_{i^{*}}\right)\mathrm{Pr}\left\{X^{n}\in B_{n}\right\}}{P_{{\bar{X}}^{n}}\left(x_{i^{*}}\right)+e^{-n\gamma }}\right). \end{array}$$

Here, using the relation

(45) $$\begin{array}{c} P_{{\bar{X}}^{n}}\left(x_{i^{*}}\right)\mathrm{Pr}\left\{X^{n}\in B_{n}\right\}\\ \;=\left(1-e^{-n\gamma }\right)P_{{\bar{X}}^{n}}\left(x_{i^{*}}\right)\mathrm{Pr}\left\{X^{n}\in B_{n}\right\}+e^{-n\gamma }P_{{\bar{X}}^{n}}\left(x_{i^{*}}\right)\mathrm{Pr}\left\{X^{n}\in B_{n}\right\}, \end{array}$$

we obtain

(46) $$\begin{array}{c} \left(P_{{\bar{X}}^{n}}\left(x_{i^{*}}\right)+e^{-n\gamma }\right)f\left(\frac{P_{{\bar{X}}^{n}}\left(x_{i^{*}}\right)\mathrm{Pr}\left\{X^{n}\in B_{n}\right\}}{P_{{\bar{X}}^{n}}\left(x_{i^{*}}\right)+e^{-n\gamma }}\right)\\ \;\leq P_{{\bar{X}}^{n}}\left(x_{i^{*}}\right)f\left(\frac{\left(1-e^{-n\gamma }\right)P_{{\bar{X}}^{n}}\left(x_{i^{*}}\right)\mathrm{Pr}\left\{X^{n}\in B_{n}\right\}}{P_{{\bar{X}}^{n}}\left(x_{i^{*}}\right)}\right)\\ \;\;+e^{-n\gamma }f\left(\frac{e^{-n\gamma }P_{{\bar{X}}^{n}}\left(x_{i^{*}}\right)\mathrm{Pr}\left\{X^{n}\in B_{n}\right\}}{e^{-n\gamma }}\right)\\ \;=P_{{\bar{X}}^{n}}\left(x_{i^{*}}\right)f\left(\left(1-e^{-n\gamma }\right)\mathrm{Pr}\left\{X^{n}\in B_{n}\right\}\right)\\ \;\;+e^{-n\gamma }f\left(P_{{\bar{X}}^{n}}\left(x_{i^{*}}\right)\mathrm{Pr}\left\{X^{n}\in B_{n}\right\}\right)\\ \;\leq P_{{\bar{X}}^{n}}\left(x_{i^{*}}\right)f\left(\left(1-e^{-n\gamma }\right)\mathrm{Pr}\left\{X^{n}\in B_{n}\right\}\right)\\ \;\;+e^{-n\gamma }f\left(e^{-n(\underline{H}\left(X\right)-\varepsilon )}\mathrm{Pr}\left\{X^{n}\in B_{n}\right\}\right) \end{array}$$

for sufficiently large *n*, where the first inequality is due to (11) and the last inequality is from (34) and the condition C1).

Hence, from C3) and the continuity of the function *f*, for ∀ν>0 we have

(47) $$\begin{array}{c} \left(P_{{\bar{X}}^{n}}\left(x_{i^{*}}\right)+e^{-n\gamma }\right)f\left(\frac{P_{{\bar{X}}^{n}}\left(x_{i^{*}}\right)\mathrm{Pr}\left\{X^{n}\in B_{n}\right\}}{P_{{\bar{X}}^{n}}\left(x_{i^{*}}\right)+e^{-n\gamma }}\right)\\ \;\leq P_{{\bar{X}}^{n}}\left(x_{i^{*}}\right)f\left(\mathrm{Pr}\left\{X^{n}\in B_{n}\right\}-e^{-n\gamma }\right)+\nu \\ \;\leq P_{{\bar{X}}^{n}}\left(x_{i^{*}}\right)f\left(\mathrm{Pr}\left\{X^{n}\in B_{n}\right\}\right)+2\nu \end{array}$$

for sufficiently large *n*. Therefore, noting that $P_{{\bar{X}}^{n}}\left(x_{i^{*}}\right)\leq P_{{\tilde{X}}^{n}}\left(x_{i^{*}}\right)$, from (28), (43), (44) and (47) it holds that

(48) $$\begin{array}{ccc} D_{f}\left(X^{n}\left|\right|\phi_{n}\left(U_{M_{n}}\right)\right) & \leq & \sum_{i=1}^{i^{*}}P_{{\tilde{X}}^{n}}\left(x_{i}\right)f\left(\mathrm{Pr}\left\{X^{n}\in B_{n}\right\}\right)+2\nu \\ & = & f\left(\mathrm{Pr}\left\{X^{n}\in B_{n}\right\}\right)+2\nu \\ & \leq & f\left(f^{-1}\left(D\right)\right)+2\nu \\ & = & D+2\nu \end{array}$$

for sufficiently large *n*. This completes the proof of the theorem. □

**Theorem 4.**  *Under conditions C1) and C2), for any mapping $\phi_{n}$ satisfying*

(49) $$D_{f}(X^{n}\left|\right|\phi_{n}\left(U_{M_{n}}\right))\leq D,$$

*it holds that*

(50) $$\frac{1}{n}\log M_{n}\geq \frac{1}{n}H_{0}\left(1-f^{-1}\left(D\right)|X^{n}\right).$$

**Proof.**  It suffices to show the fact that the relation

(51) $$\frac{1}{n}\log M_{n}<\frac{1}{n}H_{0}\left(1-f^{-1}\left(D\right)|X^{n}\right)$$

necessarily yields

(52) $$D_{f}(X^{n}\left|\right|\phi_{n}\left(U_{M_{n}}\right))>D.$$

We here denote $H^{\prime }:=H_{0}\left(1-f^{-1}\left(D\right)|X^{n}\right)$ for short. For any fixed mapping $\phi_{n}:U_{M_{n}}\to X^{n}$, we set ${\tilde{X}}^{n}:=\phi_{n}\left(U_{M_{n}}\right)$ and

(53) $$B_{n}:=\left\{x\in X^{n}|P_{{\tilde{X}}^{n}}\left(x\right)>0\right\}.$$

Then, from the property of the mapping it must hold that

(54) $$M_{n}\geq \left|B_{n}\right|.$$

From the condition C2) the *f*-divergence between $P_{X^{n}}$ and $P_{{\tilde{X}}^{n}}$ is lower bounded by

(55) $$\begin{array}{ccc} D_{f}(X^{n}\left|\right|\phi_{n}\left(U_{M_{n}}\right)) & = & \sum_{x\in B_{n}}P_{{\tilde{X}}^{n}}\left(x\right)f\left(\frac{P_{X^{n}}\left(x\right)}{P_{{\tilde{X}}^{n}}\left(x\right)}\right)\\ & \geq & f\left(\mathrm{Pr}\{X^{n}\in B_{n}\}\right)\\ & \geq & f\left(\max_{\begin{array}{c} B_{n}\subset X^{n}\\ |B_{n}|\leq M_{n} \end{array}}\mathrm{Pr}\left\{X^{n}\in B_{n}\right\}\right)\\ & \geq & f\left(\max_{\begin{array}{c} B_{n}\subset X^{n}\\ \log|B_{n}|<H^{\prime } \end{array}}\mathrm{Pr}\left\{X^{n}\in B_{n}\right\}\right)\\ & > & f\left(1-(1-f^{-1}\left(D\right))\right)\\ & = & D, \end{array}$$

where the first inequality is due to (11), the second inequality is due to condition C1) and (54) and the third inequality is from (51). The last inequality is from the definition of the alternative expressions given in Theorem 1. This completes the proof. □

Theorems 3 and 4 show that the smooth max entropy and the inverse function of *f* have important roles in the resolvability problem with respect to *f*-divergences. From these theorems, we obtain the following theorem, which addresses the *general formula* of the optimum resolvability rate. It should be noted that because of the assumption 0≤D<f(0) and C1), we have $0<f^{-1}\left(D\right)\leq 1$.

**Theorem 5.**  *Under conditions C1)–C3), it holds that*

(56) $$\begin{array}{cc} S_{r}^{\left(f\right)}\left(D|X\right) & =\lim_{\nu ↓0}\underset{n\to \infty }{lim sup}\frac{1}{n}H_{0}\left(1-f^{-1}\left(D+\nu \right)|X^{n}\right)\\ \; & =\lim_{\nu ↓0}\underset{n\to \infty }{lim sup}\frac{1}{n}H_{0}\left(1-f^{-1}\left(D\right)+\nu |X^{n}\right). \end{array}$$

**Proof.**  We here show the first equality, because the second equality can be derived from the first inequality together with the continuity of the function $f^{-1}$.

(Direct Part:) Fix ν>0 arbitrarily. From Theorem 3, for any γ>0, there exists a mapping $\phi_{n}$ such that

(57) $$\begin{array}{ccc} \frac{1}{n}\log M_{n} & \leq & \frac{1}{n}H_{0}\left(1-f^{-1}\left(D+\nu \right)|X^{n}\right)+\gamma , \end{array}$$

and

(58) $$D_{f}(X^{n}\left|\right|\phi_{n}\left(U_{M_{n}}\right))\leq D+\nu +\gamma .$$

We here use the diagonal line argument [8]. Fix a sequence ${\left\{\gamma_{i}\right\}}_{i=1}^{\infty }$ such that $\gamma_{1}>\gamma_{2}>\cdots >0$, and we repeat the above argument as i→∞. Then, we can show that there exists a mapping $\phi_{n}$ satisfying

(59) $$\underset{n\to \infty }{lim sup}D_{f}(X^{n}\left|\right|\phi_{n}\left(U_{M_{n}}\right))\leq D+\nu ,$$

and

(60) $$\begin{array}{c} \underset{n\to \infty }{lim sup}\frac{1}{n}\log M_{n}\leq \underset{n\to \infty }{lim sup}\frac{1}{n}H_{0}\left(1-f^{-1}\left(D+\nu \right)|X^{n}\right). \end{array}$$

Here, also from the diagonal line argument with respect to ν, we obtain

(61) $$\begin{array}{c} \underset{n\to \infty }{lim sup}\frac{1}{n}\log M_{n}\leq \lim_{\nu ↓0}\underset{n\to \infty }{lim sup}\frac{1}{n}H_{0}\left(1-f^{-1}\left(D+\nu \right)|X^{n}\right). \end{array}$$

This completes the proof of the direct part.

(Converse Part:) We fixed ν>0 arbitrarily. From Theorem 4, for any mapping $\phi_{n}$ satisfying

(62) $$D_{f}(X^{n}\left|\right|\phi_{n}\left(U_{M_{n}}\right))\leq D+\nu ,$$

it holds that

(63) $$\frac{1}{n}\log M_{n}\geq \frac{1}{n}H_{0}\left(1-f^{-1}\left(D+\nu \right)|X^{n}\right).$$

Consequently, we have

(64) $$\underset{n\to \infty }{lim sup}D_{f}(X^{n}\left|\right|\phi_{n}\left(U_{M_{n}}\right))\leq D+\nu$$

and

(65) $$\underset{n\to \infty }{lim sup}\frac{1}{n}\log M_{n}\geq \underset{n\to \infty }{lim sup}\frac{1}{n}H_{0}\left(1-f^{-1}\left(D+\nu \right)|X^{n}\right).$$

We also use the diagonal line argument [8]. We repeat the above argument as i→∞ for a sequence ${\left\{\nu_{i}\right\}}_{i=1}^{\infty }$ such that $\nu_{1}>\nu_{2}>\cdots >0$. Then, for any mapping $\phi_{n}$ satisfying

(66) $$\underset{n\to \infty }{lim sup}D_{f}(X^{n}\left|\right|\phi_{n}\left(U_{M_{n}}\right))\leq D,$$

it holds that

(67) $$\begin{array}{ccc} \underset{n\to \infty }{lim sup}\frac{1}{n}\log M_{n} & \geq & \lim_{\nu ↓0}\underset{n\to \infty }{lim sup}\frac{1}{n}H_{0}\left(1-f^{-1}\left(D+\nu \right)|X^{n}\right). \end{array}$$

This completes the proof of the converse part. □

## 4. Intrinsic Randomness Problem

**Figure 4:**
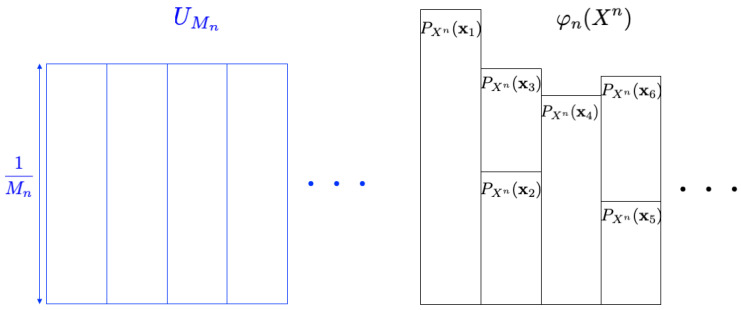
Intrinsic randomness problem.

In the previous section, we reveal the *general formula* for the optimum resolvability rate. In this section, we consider how to approximate the uniform random number $U_{M_{n}}$ by using the given discrete source $X={\left\{X^{n}\right\}}_{n=1}^{\infty }$ and the mapping $\varphi_{n}$. Figure 4 is an illustrative figure of the problem (the probability distribution for $U_{M_{n}}$ is depicted in blue, while the one for $\varphi_{n}\left(X^{n}\right)$ is shown in black). The size of the random number $M_{n}$ is requested to be as large as possible. In the intrinsic randomness problem, one of our main concerns is to derive the largest value of *b* in the form of $M_{n}=e^{nb}$ under some approximation measure [7]. This problem setting is formulated as follows.

**Definition 5.**  *R is said to be Δ-achievable with the given f-divergence if there exists a sequence of mapping $\varphi_{n}:X^{n}\to U_{M_{n}}$ such that*

(68) $$\begin{array}{ccc} \underset{n\to \infty }{lim sup}D_{f}(\varphi_{n}\left(X^{n}\right)\left|\right|U_{M_{n}}) & \leq & \Delta , \end{array}$$

(69) $$\begin{array}{ccc} \underset{n\to \infty }{lim inf}\frac{1}{n}\log M_{n} & \geq & R. \end{array}$$

In this case, given Δ, if the rate constraint *R* is sufficiently small, it can be shown that there exists a sequence of mappings that satisfies the constraints. On the other hand, if *R* is too large, no sequence of mappings that achieves the desired constraints can be found. Consequently, in this setting, the supremum of *R* is of particular interest.

**Definition 6** (First-order optimum intrinsic randomness rate).
(70) $$S_{\iota }^{\left(f\right)}\left(\Delta |X\right):=\sup\left\{R\left|R\;is\;\Delta -achievable\;with\;the\;given\;f-divergence\right.\right\}.$$

**Remark 8.**  *It should be emphasized that we use the f-divergence of the form $D_{f}(\varphi_{n}\left(X^{n}\right)\left|\right|U_{M_{n}})$ instead of $D_{f}(U_{M_{n}}\left|\right|\varphi_{n}\left(X^{n}\right))$ (cf. Remark 6).*

We also assume that Δ∈[0,f(0)) in this section (cf. Remark 7). In order to analyze the general formula of the optimum intrinsic randomness rate $S_{\iota }^{\left(f\right)}\left(\Delta |X\right)$, we first give two theorems.

**Theorem 6.**  *Under conditions C1) and C2), for any γ>0 and $M_{n}$ satisfying*

(71) $$\frac{1}{n}\log M_{n}\leq \frac{1}{n}H_{\infty }\left(1-f^{-1}\left(\Delta \right)|X^{n}\right)-\gamma ,$$

*there exists a mapping $\varphi_{n}$ such that*

(72) $$D_{f}(\varphi_{n}\left(X^{n}\right)\left|\right|U_{M_{n}})\leq \Delta +\gamma$$

*for sufficiently large n.*

**Proof.**  We set $\beta_{0}$ as

(73) $$-\log\beta_{0}=H_{\infty }\left(1-f^{-1}\left(\Delta \right)|X^{n}\right)$$

for short.

From Theorem 2, we notice that

(74) $$1-f^{-1}\left(\Delta \right)\geq \sum_{x\in X^{n}}{\left(P_{X^{n}}\left(x\right)-\beta_{0}\right)}^{+}=:1-A_{n}\left(\Delta \right),$$

where if $\beta_{0}>1/\left|X^{n}\right|$ holds, then $f^{-1}\left(\Delta \right)=A_{n}\left(\Delta \right)$. We shall show that for any $M_{n}$ satisfying

(75) $$\frac{1}{n}\log M_{n}\leq -\frac{1}{n}\log\beta_{0}-\frac{1}{n}\log\frac{1}{A_{n}\left(\Delta \right)}-\frac{\gamma }{2},$$

there exists a mapping $\varphi_{n}$ such that

(76) $$D_{f}(\varphi_{n}\left(X^{n}\right)\left|\right|U_{M_{n}})\leq \Delta +\gamma$$

for sufficiently large *n*.

For every sequence $x\in X^{n}$, we define the probability distribution

(77) $$P_{{\bar{X}}^{n}}\left(x\right):=\left\{\begin{array}{cc} \frac{\beta_{0}}{A_{n}\left(\Delta \right)} & P_{X^{n}}\left(x\right)\geq \beta_{0},\\ \frac{P_{X^{n}}\left(x\right)}{A_{n}\left(\Delta \right)} & P_{X^{n}}\left(x\right)<\beta_{0}. \end{array}\right.$$

Since $0<A_{n}\left(\Delta \right)<1$, this probability distribution is well-defined. Then, from the definition of the smooth min entropy it holds that

(78) $$\sum_{x\in X^{n}}P_{{\bar{X}}^{n}}\left(x\right)=1.$$

Here, from (75) and the definition of the smooth min entropy it holds that

(79) $$\begin{array}{cc} M_{n} & \leq \frac{1}{\beta_{0}}A_{n}\left(\Delta \right)e^{-n\gamma /2}\leq \left|X^{n}\right| \end{array}$$

for sufficiently large *n*.

We next define the mapping $\varphi_{n}:X^{n}\to U_{M_{n}}$ by using $P_{{\bar{X}}^{n}}$. To do so, we classify the elements of $X^{n}$ into $I_{n}\left(i\right)\;\left(1\leq i\leq M_{n}\right)$ as follows.

1. We choose a set $I_{n}\left(1\right)$ arbitrarily satisfying
  (80) $$\sum_{x\in I_{n}\left(1\right)}P_{{\bar{X}}^{n}}\left(x\right)\leq \frac{1}{M_{n}},$$
  (81) $$\sum_{x\in I_{n}\left(1\right)}P_{{\bar{X}}^{n}}\left(x\right)+P_{{\bar{X}}^{n}}\left(x^{\prime }\right)>\frac{1}{M_{n}}$$
  for any $x^{\prime }\in X^{n}\setminus I_{n}\left(1\right)$.
2. Next, we choose a set $I_{n}\left(2\right)\subset X^{n}\setminus I_{n}\left(1\right)$ satisfying
  (82) $$\sum_{x\in I_{n}\left(2\right)}P_{{\bar{X}}^{n}}\left(x\right)\leq \frac{1}{M_{n}},$$
  (83) $$\sum_{x\in I_{n}\left(2\right)}P_{{\bar{X}}^{n}}\left(x\right)+P_{{\bar{X}}^{n}}\left(x^{\prime }\right)>\frac{1}{M_{n}}$$
  for any $x^{\prime }\in X^{n}\setminus {⋃}_{i=1}^{2}I_{n}\left(i\right)$.

Furthermore, we repeat this operation $\left(M_{n}-1\right)$ times so as to choose sets $I_{n}\left(i\right)\;\left(1\leq i\leq M_{n}-1\right)$. Notice here that since $\frac{1}{M_{n}}>\frac{\beta_{0}}{A_{n}\left(\Delta \right)}$ holds, we can repeat this operation $\left(M_{n}-1\right)$ times. Thus, from the above procedure, all of $I_{n}\left(i\right)\;\left(1\leq i\leq M_{n}-1\right)$ are not empty. Lastly, we set $I_{n}\left(M_{n}\right)=\left\{x\in X^{n}|x\in X^{n}\setminus \cup_{i=1}^{M_{n}-1}I_{n}\left(i\right)\right)\}$.

From $I_{n}\left(i\right)\;\left(1\leq i\leq M_{n}\right)$, we define the mapping $\varphi_{n}:X^{n}\to U_{M_{n}}$ as follows:

(84) $$\varphi_{n}\left(x\right)=i,\;x\in I_{n}\left(i\right).$$

Furthermore, we set ${\tilde{U}}_{M_{n}}=\varphi_{n}\left(X^{n}\right)$. Thus,

(85) $$P_{{\tilde{U}}_{M_{n}}}\left(i\right)=\sum_{x\in I_{n}\left(i\right)}P_{X^{n}}\left(x\right)$$

holds for every *i* in $1\leq i\leq M_{n}$.

We next evaluate the above mapping $\varphi_{n}$. From the construction of the mapping, it holds that

(86) $$\frac{1}{M_{n}}<\sum_{x\in I_{n}\left(i\right)}P_{{\bar{X}}^{n}}\left(x\right)+\frac{\beta_{0}}{A_{n}\left(\Delta \right)}$$

for all $i\;(1\leq i\leq M_{n}-1)$ and

(87) $$\frac{1}{M_{n}}\leq \sum_{x\in I_{n}\left(M_{n}\right)}P_{{\bar{X}}^{n}}\left(x\right).$$

Hence, for all $i\;(1\leq i\leq M_{n})$, we have

(88) $$\frac{1}{M_{n}}-\frac{\beta_{0}}{A_{n}\left(\Delta \right)}\leq \sum_{x\in I_{n}\left(i\right)}P_{{\bar{X}}^{n}}\left(x\right).$$

Here, notice that for all $x\in X^{n}$

(89) $$P_{{\bar{X}}^{n}}\left(x\right)\leq \frac{P_{X^{n}}\left(x\right)}{A_{n}\left(\Delta \right)}$$

holds from (77). Thus, we have

(90) $$\begin{array}{ccc} \frac{P_{{\tilde{U}}_{M_{n}}}\left(i\right)}{A_{n}\left(\Delta \right)} & = & \frac{\sum_{x\in I_{n}\left(i\right)}P_{X^{n}}\left(x\right)}{A_{n}\left(\Delta \right)}\\ & \geq & \sum_{x\in I_{n}\left(i\right)}P_{{\bar{X}}^{n}}\left(x\right)\\ & > & \frac{1}{M_{n}}-\frac{\beta_{0}}{A_{n}\left(\Delta \right)}\\ & = & \frac{1}{M_{n}}\left(1-\frac{M_{n}\beta_{0}}{A_{n}\left(\Delta \right)}\right)\\ & \geq & \frac{1}{M_{n}}\left(1-e^{-n\gamma /2}\right) \end{array}$$

for all $i\;(1\leq i\leq M_{n})$ where the first equality is due to (85), the first inequality is due to (89), the second inequality is due to (88), and the last inequality is due to (79). Hence, we obtain

(91) $$\begin{array}{ccc} D_{f}(\varphi_{n}\left(X^{n}\right)\left|\right|U_{M_{n}}) & = & \sum_{1\leq i\leq M_{n}}\frac{1}{M_{n}}f\left(\frac{P_{{\tilde{U}}_{M_{n}}}\left(i\right)}{\frac{1}{M_{n}}}\right)\\ & \leq & \sum_{1\leq i\leq M_{n}}\frac{1}{M_{n}}f\left(A_{n}\left(\Delta \right)\left(1-e^{-n\gamma /2}\right)\right)\\ & \leq & f\left(f^{-1}\left(\Delta \right)\right)+\delta_{n}\\ & = & \Delta +\delta_{n}, \end{array}$$

where we can choose some $\delta_{n}>0$ such that $\delta_{n}\to 0\;\left(n\to \infty \right)$, the first inequality is due to (90), and the second inequality is due to the continuity of the function *f*, (74) and C1). This completes the proof of the theorem. □

**Theorem 7.**  *Under conditions C1) and C2), for any ε>0 if the mapping $\varphi_{n}$ satisfies*

(92) $$D_{f}(\varphi_{n}\left(X^{n}\right)\left|\right|U_{M_{n}})\leq \Delta -\varepsilon ,$$

*then it holds that*

(93) $$\frac{1}{n}\log M_{n}\leq \frac{1}{n}H_{\infty }\left(1-f^{-1}\left(\Delta \right)|X^{n}\right)$$

*for sufficiently large n.*

**Proof.**  Setting

(94) $$H^{\prime }:=H_{\infty }\left(1-f^{-1}\left(\Delta \right)|X^{n}\right),$$

we only consider the case where $H^{\prime }<\left|X^{n}\right|$ holds, because $H^{\prime }=\left|X^{n}\right|$ means the trivial result. Let ε>0 be fixed arbitrarily. We show that if

(95) $$\frac{1}{n}\log M_{n}>\frac{1}{n}H^{\prime }$$

holds for infinitely many $n=n_{1},n_{2},\cdots ,$ then for any $\varphi_{n}$ it holds that

(96) $$D_{f}(\varphi_{n}\left(X^{n}\right)\left|\right|U_{M_{n}})>\Delta -\varepsilon .$$

From (95), there exists a positive constant γ satisfying

(97) $$\frac{1}{n}\log M_{n}-2\gamma >\frac{1}{n}H^{\prime }.$$

Here, for γ>0 satisfying the above inequality we set $T_{n}$ as

(98) $$\begin{array}{cc} T_{n} & :=\left\{x\in X^{n}\left|\frac{1}{n}\log\frac{1}{P_{X^{n}}\left(x\right)}\leq \frac{1}{n}H^{\prime }+\gamma \right.\right\} \end{array}$$

(99) $$\begin{array}{cc} \; & =\left\{x\in X^{n}\left|P_{X^{n}}\left(x\right)\geq e^{-(H^{\prime }+n\gamma )}\right.\right\}. \end{array}$$

Then, from the relation

(100) $$\begin{array}{c} 1\geq \sum_{x\in T_{n}}P_{X^{n}}\left(x\right)\geq \left|T_{n}\right|e^{-(H^{\prime }+n\gamma )} \end{array}$$

we have

(101) $$|T_{n}|\leq e^{H^{\prime }+n\gamma }.$$

Next, we fix $M_{n}$ and a mapping $\varphi_{n}$ satisfying (95) and set ${\tilde{U}}_{M_{n}}$ as ${\tilde{U}}_{M_{n}}=\varphi_{n}\left(X^{n}\right)$. Using $\varphi_{n}$ and $T_{n}$, we set $I_{n}$ as

(102) $$I_{n}:=\left\{i|\exists x\in T_{n},\varphi_{n}\left(x\right)=i\right\}.$$

Thus, the set $I_{n}$ is the set of index *i* constructing from at least one $x\in T_{n}$ and the set ${\left(I_{n}\right)}^{c}$ is the set of *i* constructing only from $x\in {\left(T_{n}\right)}^{c}$.

Then, from the definition of the mapping and (101), it holds that

(103) $$|I_{n}\left|\leq \right|T_{n}|\leq e^{H^{\prime }+n\gamma }.$$

On the other hand, from (97), we have

(104) $$\begin{array}{c} M_{n}>e^{H^{\prime }+2n\gamma }. \end{array}$$

This means that

(105) $$\frac{|I_{n}|}{M_{n}}\leq e^{-n\gamma }$$

holds. Hence, from the condition C2), we have

(106) $$\frac{|I_{n}|}{M_{n}}f\left(\frac{M_{n}}{|I_{n}|}\right)\to 0\;\left(n\to \infty \right).$$

From the above argument, the *f*-divergence between $\varphi_{n}\left(X^{n}\right)$ and $U_{M_{n}}$ is evaluated as

(107) $$\begin{array}{ccc} D_{f}(\varphi_{n}\left(X^{n}\right)\left|\right|U_{M_{n}}) & = & \sum_{1\leq i\leq M_{n}}\frac{1}{M_{n}}f\left(\frac{P_{{\tilde{U}}_{M_{n}}}\left(i\right)}{\frac{1}{M_{n}}}\right)\\ & = & \sum_{1\leq i\leq M_{n},i\in I_{n}}\frac{1}{M_{n}}f\left(\frac{P_{{\tilde{U}}_{M_{n}}}\left(i\right)}{\frac{1}{M_{n}}}\right)\\ & & +\sum_{1\leq i\leq M_{n},i\in {\left(I_{n}\right)}^{c}}\frac{1}{M_{n}}f\left(\frac{P_{{\tilde{U}}_{M_{n}}}\left(i\right)}{\frac{1}{M_{n}}}\right)\\ & \geq & \frac{|I_{n}|}{M_{n}}f\left(\frac{\sum_{1\leq i\leq M_{n},i\in I_{n}}P_{{\tilde{U}}_{M_{n}}}\left(i\right)}{\frac{|I_{n}|}{M_{n}}}\right)\\ & & +\frac{|{\left(I_{n}\right)}^{c}|}{M_{n}}f\left(\frac{\sum_{1\leq i\leq M_{n},i\in {\left(I_{n}\right)}^{c}}P_{{\tilde{U}}_{M_{n}}}\left(i\right)}{\frac{|{\left(I_{n}\right)}^{c}|}{M_{n}}}\right)\\ & \geq & \frac{|I_{n}|}{M_{n}}f\left(\frac{1}{\frac{|I_{n}|}{M_{n}}}\right)+\frac{|{\left(I_{n}\right)}^{c}|}{M_{n}}f\left(\frac{\sum_{1\leq i\leq M_{n},i\in {\left(I_{n}\right)}^{c}}P_{{\tilde{U}}_{M_{n}}}\left(i\right)}{\frac{|{\left(I_{n}\right)}^{c}|}{M_{n}}}\right)\\ & \geq & \frac{|I_{n}|}{M_{n}}f\left(\frac{M_{n}}{|I_{n}|}\right)+\frac{|{\left(I_{n}\right)}^{c}|}{M_{n}}f\left(\frac{\mathrm{Pr}\{X^{n}\in {\left(T_{n}\right)}^{c}\}}{\frac{|{\left(I_{n}\right)}^{c}|}{M_{n}}}\right), \end{array}$$

where the first inequality is due to (11), and the last inequality is due to the relation

(108) $$\sum_{1\leq i\leq M_{n},i\in {\left(I_{n}\right)}^{c}}P_{{\tilde{U}}_{M_{n}}}\left(i\right)\leq \mathrm{Pr}\left\{X^{n}\in {\left(T_{n}\right)}^{c}\right\}$$

and C1).

We next focus on the evaluation of the second term on the RHS of (107). From the definition of the smooth min entropy $H^{\prime }$ and Theorem 1, for any γ>0 it necessarily holds that

(109) $$\begin{array}{c} \sum_{x\in X^{n}}{\left(P_{X^{n}}\left(x\right)-e^{-(H^{\prime }+n\gamma )}\right)}^{+}\geq 1-f^{-1}\left(\Delta \right). \end{array}$$

Thus, from the definition of $T_{n}$ it holds that

(110) $$\begin{array}{ccc} \sum_{x\in T_{n}}\left(P_{X^{n}}\left(x\right)-e^{-(H^{\prime }+n\gamma )}\right) & = & \sum_{x\in X^{n}}{\left(P_{X^{n}}\left(x\right)-e^{-(H^{\prime }+n\gamma )}\right)}^{+}\\ & \geq & 1-f^{-1}\left(\Delta \right). \end{array}$$

Thus, we obtain

(111) $$\begin{array}{ccc} \mathrm{Pr}\left\{X^{n}\in T_{n}\right\} & \geq & 1-f^{-1}\left(\Delta \right), \end{array}$$

from which it holds that

(112) $$\begin{array}{cc} \mathrm{Pr}\left\{X^{n}\in {\left(T_{n}\right)}^{c}\right\} & <1-\left(1-f^{-1}\left(\Delta \right)\right)=f^{-1}\left(\Delta \right). \end{array}$$

Plugging the above inequality with (107), we obtain

(113) $$\begin{array}{ccc} D_{f}(\varphi_{n}\left(X^{n}\right)\left|\right|U_{M_{n}}) & > & \frac{|I_{n}|}{M_{n}}f\left(\frac{M_{n}}{|I_{n}|}\right)+\frac{|{\left(I_{n}\right)}^{c}|}{M_{n}}f\left(\frac{f^{-1}\left(\Delta \right)}{\frac{|{\left(I_{n}\right)}^{c}|}{M_{n}}}\right). \end{array}$$

Noticing that

(114) $$\frac{|{\left(I_{n}\right)}^{c}|}{M_{n}}>1-e^{-n\gamma },$$

from (105), for some $\delta_{n}\to 0$, we have

(115) $$\begin{array}{ccc} D_{f}(\varphi_{n}\left(X^{n}\right)\left|\right|U_{M_{n}}) & > & \left(1-e^{-n\gamma }\right)f\left(\frac{f^{-1}\left(\Delta \right)}{1-e^{-n\gamma }}\right)-\delta_{n}\\ & = & f\left(\frac{f^{-1}\left(\Delta \right)}{1-e^{-n\gamma }}\right)-e^{-n\gamma }f\left(\frac{f^{-1}\left(\Delta \right)}{1-e^{-n\gamma }}\right)-\delta_{n}\\ & = & f\left(f^{-1}\left(\Delta \right)\left(1+\gamma_{n}^{\prime }\right)\right)-2\delta_{n} \end{array}$$

for sufficiently large *n*, where we use the property (106) and the notation $\gamma_{n}^{\prime }=\frac{e^{-n\gamma }}{1-e^{-n\gamma }}$. Since $\gamma_{n}^{\prime }\to 0\;\left(n\to \infty \right)$ holds, from the continuity of the function *f*, it holds that

(116) $$\begin{array}{ccc} D_{f}(\varphi_{n}\left(X^{n}\right)\left|\right|U_{M_{n}}) & > & \Delta -3\delta_{n}\\ & \geq & \Delta -\varepsilon \end{array}$$

for $n=n_{j},n_{j+1},\cdots ,$ with some j≥1. Therefore, we obtain the theorem. □

Theorems 6 and 7 show that the smooth min entropy and the inverse function of *f* have important roles in the intrinsic randomness problem with respect to *f*-divergences, while the smooth max entropy is important in the resolvability problem. By using the above two theorems, we obtain the following theorem. It should be noted that because of the assumption 0≤Δ<f(0) and C1), we have $0<f^{-1}\left(\Delta \right)\leq 1$.

**Theorem 8.**  *Under conditions C1) and C2), it holds that*

(117) $$\begin{array}{ccc} S_{\iota }^{\left(f\right)}\left(\Delta |X\right) & = & \lim_{\nu ↓0}\underset{n\to \infty }{lim inf}\frac{1}{n}H_{\infty }\left(1-f^{-1}\left(\Delta +\nu \right)|X^{n}\right)\\ & = & \lim_{\nu ↓0}\underset{n\to \infty }{lim inf}\frac{1}{n}H_{\infty }\left(1-f^{-1}\left(\Delta \right)+\nu |X^{n}\right).\;\; \end{array}$$

**Proof.**  (Direct Part:) Fix ν>0 arbitrarily. From Theorem 6, for any γ>0 and $M_{n}$ such that

(118) $$\begin{array}{cc} \frac{1}{n}H_{\infty }\left(1-f^{-1}\left(\Delta +\nu \right)|X^{n}\right)-2\gamma & \leq \frac{1}{n}\log M_{n}\leq \frac{1}{n}H_{\infty }\left(1-f^{-1}\left(\Delta \right)|X^{n}\right)-\gamma \end{array}$$

holds, there exists a mapping $\varphi_{n}$ satisfying

(119) $$D_{f}(\varphi_{n}\left(X^{n}\right)\left|\right|U_{M_{n}})\leq \Delta +\gamma$$

for sufficiently large *n*.

Since γ>0 is arbitrarily, we obtain

(120) $$\underset{n\to \infty }{lim inf}\frac{1}{n}\log M_{n}\geq \underset{n\to \infty }{lim inf}\frac{1}{n}H_{\infty }\left(1-f^{-1}\left(\Delta +\nu \right)|X^{n}\right).$$

Here, we fix a sequence ${\left\{\nu_{i}\right\}}_{i=1}^{\infty }$ such that $\nu_{1}>\nu_{2}>\cdots >0$, and we repeat the above argument as i→∞. Then, we can show that there exists a mapping $\varphi_{n}$ satisfying

(121) $$\underset{n\to \infty }{lim inf}\frac{1}{n}\log M_{n}\geq \lim_{\nu ↓0}\underset{n\to \infty }{lim inf}\frac{1}{n}H_{\infty }\left(1-f^{-1}\left(D+\nu \right)|X^{n}\right),$$

and

(122) $$\underset{n\to \infty }{lim sup}D_{f}(\varphi_{n}\left(X^{n}\right)\left|\right|U_{M_{n}})\leq \Delta .$$

This completes the proof of the direct part of the theorem.

(Converse Part:) Fix ν>0 arbitrarily. From Theorem 7, for any mapping $\varphi_{n}$ satisfying

(123) $$D_{f}(\varphi_{n}\left(X^{n}\right)\left|\right|U_{M_{n}})\leq \Delta +\nu ,$$

it holds that

(124) $$\frac{1}{n}\log M_{n}\leq \frac{1}{n}H_{\infty }\left(1-f^{-1}\left(\Delta +2\nu \right)|X^{n}\right).$$

Thus, for any ν>0, we obtain

(125) $$\underset{n\to \infty }{lim sup}D_{f}(\varphi_{n}\left(X^{n}\right)\left|\right|U_{M_{n}})\leq \Delta +\nu ,$$

and

(126) $$\underset{n\to \infty }{lim inf}\frac{1}{n}\log M_{n}\leq \underset{n\to \infty }{lim inf}\frac{1}{n}H_{\infty }\left(1-f^{-1}\left(\Delta +2\nu \right)|X^{n}\right).$$

Noting that ν>0 is arbitrarily, we fix a sequence ${\left\{\nu_{i}\right\}}_{i=1}^{\infty }$ such that $\nu_{1}>\nu_{2}>\cdots >0$, and we repeat the above argument as i→∞. Then, we obtain

(127) $$\underset{n\to \infty }{lim sup}D_{f}(\varphi_{n}\left(X^{n}\right)\left|\right|U_{M_{n}})\leq \Delta ,$$

and

(128) $$\begin{array}{c} \underset{n\to \infty }{lim inf}\frac{1}{n}\log M_{n}\leq \lim_{\nu ↓0}\underset{n\to \infty }{lim inf}\frac{1}{n}H_{\infty }\left(1-f^{-1}\left(\Delta +\nu \right)|X^{n}\right). \end{array}$$

This completes the proof of the converse part of the theorem. □

## 5. Relaxation of Conditions C1) and C2)

Thus far, we have derived the general formulas for the optimum resolvability rate under conditions C1)–C3) and for the optimum intrinsic randomness rate under conditions C1) and C2). In this section, we relax conditions C1) and C2) to extend the class of *f*-divergence for which we can characterize these optimum rates. Hereafter, we do not consider a linear function f(t)=a(t−1) with some *a* because it always gives a trivial case where $D_{f}\left(Z|\right|\bar{Z})=0$.

We consider the following condition, which is a relaxation of C2):

C2’) The function *f* satisfies
  (129) $$\begin{array}{c} \lim_{u\to \infty }\frac{f(u)}{u}<+\infty . \end{array}$$

For function *f* satisfying condition C2’), we denote the LHS of (129) by

(130) $$\begin{array}{c} c_{f}=\lim_{u\to \infty }\frac{f(u)}{u}. \end{array}$$

We give some examples of the function f(t), which satisfies C2’) but not C2).

- f(t)=|t−1|: The *f*-divergence is variational distance, and $c_{f}=1$.
- $f\left(t\right)={\left(1-\sqrt{t}\right)}^{2}$: The *f*-divergence is the squared Hellinger distance, and $c_{f}=1$.
- $f\left(t\right)=\frac{t^{\alpha }-\alpha t-\left(1-\alpha \right)}{\alpha (\alpha -1)}$ (0<α<1): The *f*-divergence is α-divergence, and $c_{f}=\frac{1}{1-\alpha }$.

For function f(t) satisfying condition C2’), we consider its modified function

(131) $$\begin{array}{c} f_{0}\left(t\right):=f\left(t\right)+c_{f}\left(1-t\right), \end{array}$$

which is offset by $c_{f}\left(1-t\right)$. This function is called the offset function of *f*. It should be noted that under condition C2), which is a special case of C2’), it holds that $c_{f}=0$ and thus $f_{0}\left(t\right)=f\left(t\right)$ for all t≥0. We have the following lemma:

**Lemma 1.**  *Assume that the function f(t) satisfies condition C2’). Then,*

*(i)*  *the offset function $f_{0}$ satisfies conditions C1) and (C2),*
*(ii)*  *for any pair of probability distributions $P_{Z}$ and $P_{\bar{Z}}$ with the same alphabet Z, it holds that*
(132) $$\begin{array}{c} D_{f}\left(Z|\right|\bar{Z})=D_{f_{0}}\left(Z|\right|\bar{Z}). \end{array}$$

**Proof.**  It is easily verified that $f_{0}$ is a convex function with $f_{0}\left(1\right)=0$, and claim (ii) is well-known. So, here we show claim (i). By definition, it holds that

(133) $$\begin{array}{cc} \lim_{u\to \infty }\frac{f_{0}\left(u\right)}{u} & =\lim_{u\to \infty }\left(\frac{f(u)}{u}+\frac{c_{f}\left(1-u\right)}{u}\right)\\ \; & =\lim_{u\to \infty }\frac{f(u)}{u}-c_{f}=0, \end{array}$$

which indicates that $f_{0}$ satisfies condition C2).

To show C1) being held, we use the left-derivative of $f_{0}$ at t>0, denoted as

(134) $$\begin{array}{c} f_{0}^{\prime }\left(t-\right)=\lim_{h↑0}\frac{f_{0}\left(t+h\right)-f_{0}\left(t\right)}{h} \end{array}$$

(cf. [22]). Contrary to ordinary derivatives, the left-derivative at t>0 always exists for function $f_{0}$, which is continuous. To show that $f_{0}$ satisfies condition C1), it suffices to show that $f_{0}^{\prime }\left(t-\right)\leq 0$ for all t>0. Using the left-derivative $f_{0}^{\prime }\left(t-\right)$, a tangent line at t>0 can be expressed as $f_{0}^{\prime }\left(t-\right)\cdot t+b$ with some *b*, where $f_{0}^{\prime }\left(t-\right)$ and *b* correspond to the slope and intercept of this tangent line, respectively. We call this tangent line the left-tangent line at *t*. Fixing $t^{*}>0$ arbitrarily, let $a^{*}:=f_{0}^{\prime }\left(t^{*}-\right)$ and $b^{*}$ be the intercept of the left-tangent line at $t^{*}$. The convexity of $f_{0}$ implies that

(135) $$\begin{array}{c} f_{0}\left(t\right)\geq a^{*}t+b^{*}\;\;\left(\forall t\geq 0\right). \end{array}$$

Then, it follows from (133) that

(136) $$\begin{array}{c} 0=\lim_{u\to \infty }\frac{f_{0}\left(u\right)}{u}\geq \lim_{u\to \infty }\frac{a^{*}u+b^{*}}{u}=a^{*}=f_{0}^{\prime }\left(t^{*}-\right). \end{array}$$

Since $t^{*}>0$ is arbitrary, this inequality implies that $f_{0}\left(t\right)$ is decreasing for t>0 with $f_{0}\left(0\right)>0$, completing the proof of the lemma. □

Lemma 1 indicates that if the original function *f* satisfies condition C2’), then its offset function $f_{0}$ satisfies conditions C1) and C2) without changing the value of *f*-divergence. Because condition C2) is a special instance of condition C2’) with $c_{f}=0$, claim (i) of Lemma 1 implies that condition C1) is superfluous for functions satisfying C2) (cf. Remark 5). The following proposition is immediately obtained by claim (ii) of Lemma 1:

**Proposition 1.**  *Assume that the function f(t) satisfies condition C2’). Then, we have*

(137) $$\begin{array}{cc} S_{r}^{\left(f\right)}\left(D|X\right) & =S_{r}^{\left(f_{0}\right)}\left(D|X\right), \end{array}$$

(138) $$\begin{array}{cc} S_{\iota }^{\left(f\right)}\left(\Delta |X\right) & =S_{\iota }^{\left(f_{0}\right)}\left(\Delta |X\right). \end{array}$$

It is easily verified if *f* satisfies condition C3) as well as C2’), then so does $f_{0}$. From this fact, Lemma 1, and Proposition 1, we have the following generalization of Theorem 5.

**Theorem 9.**  *Under conditions C2’) and C3), it holds that*

(139) $$\begin{array}{cc} S_{r}^{\left(f\right)}\left(D|X\right) & =\lim_{\nu ↓0}\underset{n\to \infty }{lim sup}\frac{1}{n}H_{0}\left(1-f_{0}^{-1}\left(D+\nu \right)|X^{n}\right)\\ \; & =\lim_{\nu ↓0}\underset{n\to \infty }{lim sup}\frac{1}{n}H_{0}\left(1-f_{0}^{-1}\left(D\right)+\nu |X^{n}\right). \end{array}$$

For the optimum intrinsic randomness rate, we also have the generalized result of Theorem 8.

**Theorem 10.**  *Under condition C2’), it holds that*

(140) $$\begin{array}{cc} S_{\iota }^{\left(f\right)}\left(D|X\right) & =\lim_{\nu ↓0}\underset{n\to \infty }{lim inf}\frac{1}{n}H_{\infty }\left(1-f_{0}^{-1}\left(\Delta +\nu \right)|X^{n}\right)\\ \; & =\lim_{\nu ↓0}\underset{n\to \infty }{lim inf}\frac{1}{n}H_{\infty }\left(1-f_{0}^{-1}\left(\Delta \right)+\nu |X^{n}\right). \end{array}$$

## 6. Particularization to Several Distance Measures

In previous sections, we have derived the general formula of the *first-order* optimum resolvability and intrinsic randomness rates with respect to *f*-divergences, where the smooth Rényi entropy and the inverse function of *f* have important roles. In this section, we first focus on several specified functions *f* satisfying conditions C1)–C3) and compute these rates by using Theorems 5 and 8. In addition, we consider the function *f* satisfying C2’) and C3) and compute the rates by using Theorems 9 and 10.

It will turn out that it is easy to derive the optimum achievable rates for specified approximation measures. We use the notation

(141) $$D_{f}(X^{n}\left|\right|{\tilde{X}}^{n}):=D_{f}(X^{n}\left|\right|\phi_{n}\left(U_{M_{n}}\right)),\;D_{f}({\tilde{U}}_{M_{n}}\left|\right|U_{M_{n}}):=D_{f}(\varphi_{n}\left(X^{n}\right)\left|\right|U_{M_{n}})$$

for convenience.

**Remark 9.**  *Since the function f(t)=tlogt (which indicates the KL divergence) does not satisfy C1) and C2), we can not apply Theorems 5 and 8 to the case of the KL divergence:*

(142) $$\begin{array}{ccc} D_{f}(X^{n}\left|\right|{\tilde{X}}^{n})=D(X^{n}\left|\right|{\tilde{X}}^{n}) & = & \sum_{x\in X^{n}}P_{X^{n}}\left(x\right)\log\frac{P_{X^{n}}\left(x\right)}{P_{{\tilde{X}}^{n}}\left(x\right)}, \end{array}$$

(143) $$\begin{array}{ccc} D_{f}({\tilde{U}}_{M_{n}}\left|\right|U_{M_{n}})=D({\tilde{U}}_{M_{n}}\left|\right|U_{M_{n}}) & = & \sum_{1\leq i\leq M_{n}}P_{{\tilde{U}}_{M_{n}}}\left(i\right)\log\frac{P_{{\tilde{U}}_{M_{n}}}\left(i\right)}{P_{U_{M_{n}}}\left(i\right)}. \end{array}$$

*The resolvability problem with respect to the KL divergence of this direction has not been considered yet. On the other hand, in the intrinsic randomness problem, Hayashi [9] ([Theorem 7]) has studied the problem with respect to the normalized KL divergence: $1/nD({\tilde{U}}_{M_{n}}||U_{M_{n}})$ as well as $D(U_{M_{n}}||{\tilde{U}}_{M_{n}})$.*

### 6.1. Half Variational Distance

We first consider the case of f(t) given as $f\left(t\right)={\left(1-t\right)}^{+}$, which indicates

(144) $$\begin{array}{ccc} D_{f}(X^{n}\left|\right|{\tilde{X}}^{n}) & = & \frac{1}{2}\sum_{x\in X^{n}}\left|P_{{\tilde{X}}^{n}}\left(x\right)-P_{X^{n}}\left(x\right)\right|, \end{array}$$

(145) $$\begin{array}{ccc} D_{f}({\tilde{U}}_{M_{n}}\left|\right|U_{M_{n}}) & = & \frac{1}{2}\sum_{1\leq i\leq M_{n}}\left|P_{{\tilde{U}}_{M_{n}}}\left(i\right)-P_{U_{M_{n}}}\left(i\right)\right|. \end{array}$$

In this special case, we obtain the following corollary:

**Corollary 1.**  *For $f\left(t\right)={\left(1-t\right)}^{+}$, it holds that*

(146) $$\begin{array}{ccc} S_{r}^{\left(f\right)}\left(D|X\right) & = & \lim_{\nu ↓0}\underset{n\to \infty }{lim sup}\frac{1}{n}H_{0}\left(D+\nu |X^{n}\right), \end{array}$$

(147) $$\begin{array}{ccc} S_{\iota }^{\left(f\right)}\left(\Delta |X\right) & = & \lim_{\nu ↓0}\underset{n\to \infty }{lim inf}\frac{1}{n}H_{\infty }\left(\Delta +\nu |X^{n}\right). \end{array}$$

**Proof.**  In the case of $f\left(t\right)={\left(1-t\right)}^{+}$, the inverse function becomes $f^{-1}\left(D\right)=1-D$, because 0≤D<1 holds. Hence, from Theorems 5 and 8, we obtain the corollary. □

The former result in the above corollary coincides with the result given by Uyematsu [6] ([Theorem 6]), while the latter one coincides with the result given by Uyematsu and Kunimatsu [10] ([Theorem 6]). It is important to note that $S_{r}^{\left(f\right)}\left(D|X\right)$ has been addressed by Steinberg [2] and Han [8] ([Theorem 2.4.1]), and $S_{\iota }^{\left(f\right)}\left(D|X\right)$ has also been addressed by Vembu and Verdú [7] ([Theorem 1]), Han [8] ([Theorem 2.4.2]), and Hayashi [9] ([Theorem 2]), using different information-theoretic approaches. In particular, Hayashi [9] ([Theorem 2]) has considered various achievable rates concerning the intrinsic randomness problem with respect to the variational distance, but these are not included in our current analysis. Our work provides an alternative derivation and contextualizes these results within our framework of *f*-divergences.

### 6.2. Reverse Kullback–Leibler Divergence

Secondly, we consider the case of f(t)=−logt, which indicates

(148) $$\begin{array}{ccc} D_{f}(X^{n}\left|\right|{\tilde{X}}^{n}) & = & D(\phi_{n}\left(U_{M_{n}}\right)\left|\right|X^{n})=\sum_{x\in X^{n}}P_{{\tilde{X}}^{n}}\left(x\right)\log\frac{P_{{\tilde{X}}^{n}}\left(x\right)}{P_{X^{n}}\left(x\right)}, \end{array}$$

(149) $$\begin{array}{ccc} D_{f}({\tilde{U}}_{M_{n}}\left|\right|U_{M_{n}}) & = & D(U_{M_{n}}\left|\right|\varphi_{n}\left(X^{n}\right))=\sum_{1\leq i\leq M_{n}}P_{U_{M_{n}}}\left(i\right)\log\frac{P_{U_{M_{n}}}\left(i\right)}{P_{{\tilde{U}}_{M_{n}}}\left(i\right)}. \end{array}$$

In this case, we obtain the following corollary:

**Corollary 2.**  *For f(t)=−logt, it holds that*

(150) $$\begin{array}{ccc} S_{r}^{\left(f\right)}\left(D|X\right) & = & \lim_{\nu ↓0}\underset{n\to \infty }{lim sup}\frac{1}{n}H_{0}\left(1-e^{-(D+\nu )}|X^{n}\right), \end{array}$$

(151) $$\begin{array}{ccc} S_{\iota }^{\left(f\right)}\left(\Delta |X\right) & = & \lim_{\nu ↓0}\underset{n\to \infty }{lim inf}\frac{1}{n}H_{\infty }\left(1-e^{-(\Delta +\nu )}|X^{n}\right). \end{array}$$

**Proof.**  The inverse function is immediately given by $f^{-1}\left(D\right)=e^{-D}$. Hence, from Theorems 5 and 8, we obtain the corollary. □

It is important to note that $S_{\iota }^{\left(f\right)}\left(D|X\right)$ has been previously addressed by Hayashi [9] ([Theorem 7]) using different information-theoretic approaches. In particular, Vembu and Verdú [7] ([Theorem 1]) and Hayashi [9] ([Theorem 7]) have also considered the intrinsic randomness problem with respect to the normalized KL divergence, which is not included in our current analysis.

### 6.3. Hellinger Distance

We consider the case of $f\left(t\right)=1-\sqrt{t}$, which indicates

(152) $$\begin{array}{ccc} D_{f}(X^{n}\left|\right|{\tilde{X}}^{n}) & = & 1-\sum_{x\in X^{n}}\sqrt{P_{X^{n}}\left(x\right)P_{{\tilde{X}}^{n}}\left(x\right)}, \end{array}$$

(153) $$\begin{array}{ccc} D_{f}({\tilde{U}}_{M_{n}}\left|\right|U_{M_{n}}) & = & 1-\sum_{1\leq i\leq M_{n}}\sqrt{P_{{\tilde{U}}_{M_{n}}}\left(i\right)P_{U_{M_{n}}}\left(i\right)}. \end{array}$$

In this case, we obtain the following corollary:

**Corollary 3.**  *For $f\left(t\right)=1-\sqrt{t}$, it holds that*

(154) $$\begin{array}{ccc} S_{r}^{\left(f\right)}\left(D|X\right) & = & \lim_{\nu ↓0}\underset{n\to \infty }{lim sup}\frac{1}{n}H_{0}\left(2D-D^{2}+\nu |X^{n}\right), \end{array}$$

(155) $$\begin{array}{ccc} S_{\iota }^{\left(f\right)}\left(\Delta |X\right) & = & \lim_{\nu ↓0}\underset{n\to \infty }{lim inf}\frac{1}{n}H_{\infty }\left(2\Delta -\Delta^{2}+\nu |X^{n}\right). \end{array}$$

**Proof.**  The inverse function of $f\left(t\right)=1-\sqrt{t}$ is given by $f^{-1}\left(D\right)={\left(1-D\right)}^{2}$. Hence, from Theorems 5 and 8, we obtain the corollary. Notice here that since both of *D* and Δ are smaller than one, $2D-D^{2}$ as well as $2\Delta -\Delta^{2}$ are positive. □

It is worth noting that Kumagai and Hayashi [13] have analyzed this quantity for the case of i.i.d. sources. Importantly, they addressed this quantity as part of a broader problem: the random number conversion problem. On the other hand, our approach differs in that we derive this quantity from results based on *f*-divergences.

### 6.4. E_γ_-Divergence

We consider the case of $f\left(t\right)={\left(\gamma -t\right)}^{+}+1-\gamma$, which indicates

(156) $$\begin{array}{ccc} D_{f}(X^{n}\left|\right|{\tilde{X}}^{n}) & = & \sum_{x\in X^{n}:P_{X^{n}}\left(x\right)>\gamma P_{{\tilde{X}}^{n}}\left(x\right)}\left(P_{X^{n}}\left(x\right)-\gamma P_{{\tilde{X}}^{n}}\left(x\right)\right). \end{array}$$

(157) $$\begin{array}{ccc} D_{f}({\tilde{U}}_{M_{n}}\left|\right|U_{M_{n}}) & = & \sum_{1\leq i\leq M_{n}:P_{{\tilde{U}}_{M_{n}}}\left(i\right)>\gamma P_{U_{M_{n}}}\left(i\right)}\left(P_{{\tilde{U}}_{M_{n}}}\left(i\right)-\gamma P_{U_{M_{n}}}\left(i\right)\right). \end{array}$$

In this case, we obtain the corollary:

**Corollary 4.**  *For $f\left(t\right)={\left(\gamma -t\right)}^{+}+1-\gamma$, we have*

(158) $$\begin{array}{ccc} S_{r}^{\left(f\right)}\left(D|X\right) & = & \lim_{\nu ↓0}\underset{n\to \infty }{lim sup}\frac{1}{n}H_{0}\left(D+\nu |X^{n}\right), \end{array}$$

(159) $$\begin{array}{ccc} S_{\iota }^{\left(f\right)}\left(\Delta |X\right) & = & \lim_{\nu ↓0}\underset{n\to \infty }{lim inf}\frac{1}{n}H_{\infty }\left(\Delta +\nu |X^{n}\right). \end{array}$$

**Proof.**  Noting that γ≥1, we have f(t)=1−t. Hence, the corollary holds. □

**Remark 10.**  *The above corollary shows that both optimum achievable rates with respect to the $E_{\gamma }$-divergence does not depend on γ, which means that these rates coincide with the optimum achievable rates with respect to the half variational distance (cf. Corollary 1).*

### 6.5. Variational Distance

We next consider functions *f* satisfying C2’) and C3). Firstly, the function f(t)=|1−t| is considered:

(160) $$\begin{array}{ccc} D_{f}(X^{n}\left|\right|{\tilde{X}}^{n}) & = & \sum_{x\in X^{n}}\left|P_{{\tilde{X}}^{n}}\left(x\right)-P_{X^{n}}\left(x\right)\right|, \end{array}$$

(161) $$\begin{array}{ccc} D_{f}({\tilde{U}}_{M_{n}}\left|\right|U_{M_{n}}) & = & \sum_{1\leq i\leq M_{n}}\left|P_{{\tilde{U}}_{M_{n}}}\left(i\right)-P_{U_{M_{n}}}\left(i\right)\right|. \end{array}$$

As we have already mentioned in the previous section, f(t)=|1−t| does not satisfy C1). However, it satisfies C2’) and C3). Hence, from Theorems 9 and 10, we obtain the corollary:

**Corollary 5.**  *For f(t)=|1−t|, we have*

(162) $$\begin{array}{ccc} S_{r}^{\left(f\right)}\left(D|X\right) & = & \lim_{\nu ↓0}\underset{n\to \infty }{lim sup}\frac{1}{n}H_{0}\left(\left.\frac{D}{2}+\nu \right|X^{n}\right), \end{array}$$

(163) $$\begin{array}{ccc} S_{\iota }^{\left(f\right)}\left(\Delta |X\right) & = & \lim_{\nu ↓0}\underset{n\to \infty }{lim inf}\frac{1}{n}H_{\infty }\left(\left.\frac{\Delta }{2}+\nu \right|X^{n}\right). \end{array}$$

**Proof.**  Noticing that $c_{f}=1$, we have $f_{0}\left(t\right)=\left|1-t\right|+\left(1-t\right)$, from which we obtain

(164) $$f_{0}^{-1}\left(D\right)=1-\frac{D}{2}.$$

Therefore, we obtain the corollary from Theorems 9 and 10. □

### 6.6. Squared Hellinger Distance

We consider the function $f\left(t\right)={\left(1-\sqrt{t}\right)}^{2}$, which is also satisfies C2’) and C3). It indicates

(165) $$\begin{array}{ccc} D_{f}(X^{n}\left|\right|{\tilde{X}}^{n}) & = & \sum_{x\in X^{n}}{\left(\sqrt{P_{{\tilde{X}}^{n}}\left(x\right)}-\sqrt{P_{X^{n}}\left(x\right)}\right)}^{2}, \end{array}$$

(166) $$\begin{array}{ccc} D_{f}({\tilde{U}}_{M_{n}}\left|\right|U_{M_{n}}) & = & \sum_{1\leq i\leq M_{n}}{\left(\sqrt{P_{{\tilde{U}}_{M_{n}}}\left(i\right)}-\sqrt{P_{U_{M_{n}}}\left(i\right)}\right)}^{2}. \end{array}$$

In this case, we also apply Theorems 9 and 10.

**Corollary 6.**  *For $f\left(t\right)={\left(1-\sqrt{t}\right)}^{2}$, we have*

(167) $$\begin{array}{ccc} S_{r}^{\left(f\right)}\left(D|X\right) & = & \lim_{\nu ↓0}\underset{n\to \infty }{lim sup}\frac{1}{n}H_{0}\left(\left.D-\frac{D^{2}}{4}+\nu \right|X^{n}\right), \end{array}$$

(168) $$\begin{array}{ccc} S_{\iota }^{\left(f\right)}\left(\Delta |X\right) & = & \lim_{\nu ↓0}\underset{n\to \infty }{lim inf}\frac{1}{n}H_{\infty }\left(\left.D-\frac{D^{2}}{4}+\nu \right|X^{n}\right). \end{array}$$

**Proof.**  Noticing that $c_{f}=1$, we obtain

(169) $$f_{0}^{-1}\left(D\right)={\left(1-\frac{D}{2}\right)}^{2}.$$

Hence, we obtain the corollary. □

**Remark 11.**  *The variational distance is twice the half variational distance. Consequently, the results of Corollary 5 can be trivially derived from those of Corollary 1. However, we emphasize that f(t)=|1−t| does not satisfy conditions C1) and C2). Therefore, to derive the results of Corollary 5, it is necessary to apply the discussion from Section 5, specifically the examination using $f_{0}\left(t\right)$. This underscores the importance of our theoretical framework in handling cases where the function f does not meet conditions C1) and C2). A similar relationship exists between Corollary 3 and the later-discussed Corollary 6.*

### 6.7. *α*-Divergence

We consider the function $f\left(t\right)=\frac{t^{\alpha }-\alpha t-\left(1-\alpha \right)}{\alpha (\alpha -1)}$ (0<α<1), which also satisfies C2’) and C3). The α-divergence in our setting is given by

(170) $$\begin{array}{ccc} D_{f}(X^{n}\left|\right|{\tilde{X}}^{n}) & = & \frac{1}{\alpha (1-\alpha )}\left(1-\sum_{x\in X^{n}}P_{X^{n}}{\left(x\right)}^{\alpha }P_{{\tilde{X}}^{n}}{\left(x\right)}^{1-\alpha }\right), \end{array}$$

(171) $$\begin{array}{ccc} D_{f}({\tilde{U}}_{M_{n}}\left|\right|U_{M_{n}}) & = & \frac{1}{\alpha (1-\alpha )}\left(1-\sum_{1\leq i\leq M_{n}}P_{{\tilde{U}}_{M_{n}}}{\left(i\right)}^{\alpha }P_{U_{M_{n}}}{\left(i\right)}^{1-\alpha }\right). \end{array}$$

In this case, we obtain the following corollary using Theorems 9 and 10.

**Corollary 7.**  *For $f\left(t\right)=\frac{t^{\alpha }-\alpha t-\left(1-\alpha \right)}{\alpha (\alpha -1)}$, we have*

(172) $$\begin{array}{ccc} S_{r}^{\left(f\right)}\left(D|X\right) & = & \lim_{\nu ↓0}\underset{n\to \infty }{lim sup}\frac{1}{n}H_{0}\left(\left.1-{\left(D\alpha (\alpha -1)+1\right)}^{1/\alpha }+\nu \right|X^{n}\right), \end{array}$$

(173) $$\begin{array}{ccc} S_{\iota }^{\left(f\right)}\left(\Delta |X\right) & = & \lim_{\nu ↓0}\underset{n\to \infty }{lim inf}\frac{1}{n}H_{\infty }\left(\left.1-{\left(D\alpha (\alpha -1)+1\right)}^{1/\alpha }+\nu \right|X^{n}\right). \end{array}$$

**Proof.**  Noticing that $c_{f}=1/\left(1-\alpha \right)$, we obtain

(174) $$\begin{array}{ccc} f_{0}\left(t\right) & = & \frac{t^{\alpha }-1}{\alpha (\alpha -1)}\Leftrightarrow f_{0}^{-1}\left(D\right)={\left(D\alpha (\alpha -1)+1\right)}^{1/\alpha }. \end{array}$$

Hence, we obtain the corollary. □

Let us consider the case of α=1/2. In this case, the inverse function can be simply expressed as

(175) $$f_{0}^{-1}\left(D\right)={\left(1-\frac{D}{4}\right)}^{2}.$$

Hence, we have

(176) $$\begin{array}{ccc} S_{r}^{\left(f\right)}\left(D|X\right) & = & \lim_{\nu ↓0}\underset{n\to \infty }{lim sup}\frac{1}{n}H_{0}\left(\left.\frac{D}{2}-\frac{D^{2}}{16}+\nu \right|X^{n}\right). \end{array}$$

It is known that α-divergence with α=1/2 is related to the squared Hellinger distance. In actuality, the optimum resolvability rate $S_{r}^{\left(f\right)}\left(D|X\right)$ with respect to the squared Hellinger distance is identical to $S_{r}^{\left(f\right)}\left(2D|X\right)$ with respect to the α-divergence with α=1/2.

## 7. Second-Order Optimum Achievable Rate

Thus far, we have considered the *first-order* optimum resolvability rate as well as the *first-order* optimum intrinsic randomness rate. The rate of the *second-order*, which enables us to make a finer evaluation of achievable rates, has already been investigated in several information-theoretic problems [5,9,23,24,25,26,27,28,29,30]. In this section, according to these results, we also consider the *second-order* optimum achievable rates in random number generation problems with respect to *f*-divergences.

It is important to acknowledge that the second-order analysis for information-theoretic problems was initiated by Hayashi [9]. Building upon these works, Kumagai and Hayashi [13] conducted a second-order analysis for the broader random number conversion problem. They focused on i.i.d. sources and provided a more detailed analysis in this context. On the other hand, our results apply to more general sources, including but not limited to i.i.d. sources.

### 7.1. General Formula

We first define the second-order achievability in the resolvability problem.

**Definition 7.**  *L is said to be (D,R)-achievable with the given f-divergence if there exists a sequence mapping $\phi_{n}:U_{M_{n}}\to X^{n}$ such that*

(177) $$\begin{array}{c} \underset{n\to \infty }{lim sup}D_{f}(X^{n}\left|\right|\phi_{n}\left(U_{M_{n}}\right))\leq D, \end{array}$$

(178) $$\begin{array}{c} \underset{n\to \infty }{lim sup}\frac{1}{\sqrt{n}}\log\frac{M_{n}}{e^{nR}}\leq L. \end{array}$$

**Definition 8** (Second-order optimum resolvability rate).
$$\begin{array}{c} S_{r}^{\left(f\right)}\left(D,R|X\right):=\inf\left\{L\left|L\;is\;(D,R)-achievable\;with\;the\;given\;f-divergence\right.\right\}. \end{array}$$

In order to analyze the above quantity, we use the following condition instead of C3):

**C3’)**  For any pair of positive real numbers (a,b), it holds that
  (179) $$\lim_{n\to \infty }\frac{f\left(e^{-nb}\right)}{e^{\sqrt{n}a}}=0.$$

Here, functions f(t)=−logt, $f\left(t\right)=1-\sqrt{t}$, $f\left(t\right)={\left(1-t\right)}^{+}$, and $f\left(t\right)={\left(\gamma -t\right)}^{+}+\left(1-\gamma \right)$ satisfy the condition C3’). Then, the following theorem holds:

**Theorem 11** (Second-order optimum resolvability rate). *Under conditions C2’) and C3’), it holds that*

(180) $$\begin{array}{cc} S_{r}^{\left(f\right)}\left(D,R|X\right) & =\lim_{\nu ↓0}\underset{n\to \infty }{lim sup}\frac{H_{0}\left(1-f_{0}^{-1}\left(D+\nu \right)|X^{n}\right)-nR}{\sqrt{n}}, \end{array}$$

*where $f_{0}$ is the offset function of f, defined in (131).*

*In particular, under conditions C2) and C3’), it holds that*

(181) $$\begin{array}{cc} S_{r}^{\left(f\right)}\left(D,R|X\right) & =\lim_{\nu ↓0}\underset{n\to \infty }{lim sup}\frac{H_{0}\left(1-f^{-1}\left(D+\nu \right)|X^{n}\right)-nR}{\sqrt{n}}. \end{array}$$

**Proof.**  Noticing that Lemma 1 indicates that the offset function $f_{0}$ satisfies conditions C1)–C3), the proof of (180) proceeds in parallel with proofs of Theorems 3–5 in which *f*, $\frac{1}{n}$ and $e^{-n\gamma }$ are replaced by $f_{0}$, $\frac{1}{\sqrt{n}}$ and $e^{-\sqrt{n}\gamma }$, respectively. Equation (181) is a special case of (180) with $f_{0}=f$. □

We next consider the case of the intrinsic randomness problem.

**Definition 9.**  *L is said to be (Δ,R)-achievable with the given f-divergence if there exists a sequence of mapping $\varphi_{n}:X^{n}\to U_{M_{n}}$ such that*

(182) $$\begin{array}{cc} \underset{n\to \infty }{lim sup}D_{f}(\varphi_{n}\left(X^{n}\right)\left|\right|U_{M_{n}}) & \leq \Delta , \end{array}$$

(183) $$\begin{array}{cc} \underset{n\to \infty }{lim inf}\frac{1}{\sqrt{n}}\log\frac{M_{n}}{e^{nR}} & \geq L. \end{array}$$

**Definition 10** (Second-order optimum intrinsic randomness rate).
(184) $$S_{\iota }^{\left(f\right)}\left(\Delta ,R|X\right):=\sup\left\{L\left|L\;is\;(\Delta ,R)-achievable\;with\;the\;given\;f-divergence\right.\right\}.$$

Then, we have the theorem:

**Theorem 12**  (Second-order optimum intrinsic randomness rate). *Under condition C2’), it holds that*

(185) $$\begin{array}{c} S_{\iota }^{\left(f\right)}\left(\Delta ,R|X\right)=\lim_{\nu ↓0}\underset{n\to \infty }{lim inf}\frac{H_{\infty }\left(1-f_{0}^{-1}\left(\Delta +\nu \right)|X^{n}\right)-nR}{\sqrt{n}}, \end{array}$$

*where $f_{0}$ is the offset function of f.*

*In particular, under condition C2), it holds that*

(186) $$\begin{array}{c} S_{\iota }^{\left(f\right)}\left(\Delta ,R|X\right)=\lim_{\nu ↓0}\underset{n\to \infty }{lim inf}\frac{H_{\infty }\left(1-f^{-1}\left(\Delta +\nu \right)|X^{n}\right)-nR}{\sqrt{n}}, \end{array}$$

**Proof.**  The proof of (185) proceeds in parallel with proofs of Theorems 6–8 in which *f* and $\frac{1}{n}$ are replaced by $f_{0}$ and $\frac{1}{\sqrt{n}}$. Equation (186) is a special case of (185) with $f_{0}=f$. □

Theorems 11 and 12 show that in both the resolvability and the intrinsic randomness, the smooth Rényi entropy and the inverse function of *f* also have essential roles to express *second-order* optimum achievable rates.

### 7.2. Particularizations to Several Distance Measures

Analogously to Section IV, we compute $S_{r}^{\left(f\right)}\left(D,R|X\right)$ and $S_{\iota }^{\left(f\right)}\left(\Delta ,R|X\right)$ for the specified function *f* satisfying C1), C2) and C3’), by using Theorems 11 and 12. We obtain the following corollary:

**Corollary 8.**  *For $f\left(t\right)={\left(1-t\right)}^{+}$, it holds that*

(187) $$\begin{array}{ccc} S_{r}^{\left(f\right)}\left(D,R|X\right) & = & \lim_{\nu ↓0}\underset{n\to \infty }{lim sup}\frac{H_{0}\left(D+\nu |X^{n}\right)-nR}{\sqrt{n}}, \end{array}$$

(188) $$\begin{array}{ccc} S_{\iota }^{\left(f\right)}\left(\Delta ,R|X\right) & = & \lim_{\nu ↓0}\underset{n\to \infty }{lim inf}\frac{H_{\infty }\left(\Delta +\nu |X^{n}\right)-nR}{\sqrt{n}}. \end{array}$$

*For f(t)=−logt, it holds that*

(189) $$\begin{array}{ccc} S_{r}^{\left(f\right)}\left(D,R|X\right) & = & \lim_{\nu ↓0}\underset{n\to \infty }{lim sup}\frac{H_{0}\left(1-e^{-(D+\nu )}|X^{n}\right)-nR}{\sqrt{n}}, \end{array}$$

(190) $$\begin{array}{ccc} S_{\iota }^{\left(f\right)}\left(\Delta ,R|X\right) & = & \lim_{\nu ↓0}\underset{n\to \infty }{lim inf}\frac{H_{\infty }\left(1-e^{-(\Delta +\nu )}|X^{n}\right)-nR}{\sqrt{n}}. \end{array}$$

*For $f\left(t\right)=1-\sqrt{t}$, it holds that*

(191) $$\begin{array}{ccc} S_{r}^{\left(f\right)}\left(D,R|X\right) & = & \lim_{\nu ↓0}\underset{n\to \infty }{lim sup}\frac{H_{0}\left(2D-D^{2}+\nu |X^{n}\right)-nR}{\sqrt{n}}, \end{array}$$

(192) $$\begin{array}{ccc} S_{\iota }^{\left(f\right)}\left(\Delta ,R|X\right) & = & \lim_{\nu ↓0}\underset{n\to \infty }{lim inf}\frac{H_{\infty }\left(2\Delta -\Delta^{2}+\nu |X^{n}\right)-nR}{\sqrt{n}}. \end{array}$$

*For $f\left(t\right)={\left(\gamma -t\right)}^{+}+1-\gamma$, we have*

(193) $$\begin{array}{ccc} S_{r}^{\left(f\right)}\left(D,R|X\right) & = & \lim_{\nu ↓0}\underset{n\to \infty }{lim sup}\frac{H_{0}\left(D+\nu |X^{n}\right)-nR}{\sqrt{n}}, \end{array}$$

(194) $$\begin{array}{ccc} S_{\iota }^{\left(f\right)}\left(\Delta ,R|X\right) & = & \lim_{\nu ↓0}\underset{n\to \infty }{lim inf}\frac{H_{\infty }\left(\Delta +\nu |X^{n}\right)-nR}{\sqrt{n}}. \end{array}$$

**Proof.**  The proof is similar to proofs of Corollaries 1–4. □

The optimum achievable rates with the variational distance in terms of the smooth Rényi entropy have already been derived. Relations (187) and (188) coincide with the result given by Tagashira and Uyematsu [31], and the result given by Namekawa and Uyematsu [32], respectively. As in the case of the *first-order* achievability, *second-order* optimum rates for the half variational distance ($f\left(t\right)={\left(1-t\right)}^{+}$) and the $E_{\gamma }$-divergence ($f\left(t\right)={\left(\gamma -t\right)}^{+}+1-\gamma$) are the same, regardless of the value of γ≥1.

It is important to note that $S_{\iota }^{\left(f\right)}\left(\Delta ,R|X\right)$ in the case of the variational distance and the reverse KL divergence has been addressed by Hayashi [9] ([Theorem 3]) and [9] ([Theorem 9]), respectively, using different information-theoretic approaches. Furthermore, $S_{\iota }^{\left(f\right)}\left(\Delta ,R|X\right)$, in the case of the Hellinger distance for the i.i.d. case, were studied by Kumagai and Hayashi [13] for the broader setting: the random number conversion problem. Their work focused specifically on i.i.d. sources, while our results extend to more general source models.

## 8. Optimistic Optimum Achievable Rates

### 8.1. Source Resolvability

In previous sections, we have treated general formulas of the first- and second-order optimum rates. In this section, we consider optimum achievable rates in the optimistic sense. The notion of the optimistic optimum rates was first introduced by Vembu, Verdú and Steinberg [33] in the source-channel coding problem. Several researchers have developed an optimistic coding scenario in other information-theoretic problems [4,9,34,35]. In this subsection, we also clarify the optimistic optimum resolvability rate with respect to the *f*-divergence using the smooth Rényi entropy.

**Definition 11.**  *R is said to be optimistically D-achievable with the given f-divergence if there exists a sequence of mapping $\phi_{n}:U_{M_{n}}\to X^{n}$ satisfying*

(195) $$\underset{n\to \infty }{lim sup}D_{f}(X^{n}\left|\right|\phi_{n}\left(U_{M_{n}}\right))\leq D,\;\underset{n\to \infty }{lim inf}\frac{1}{n}\log M_{n}\leq R.$$

**Definition 12** (Optimistic first-order optimum resolvability rate).
(196) $$\begin{array}{cc} T_{r}^{\left(f\right)}\left(D|X\right) & :=\inf\left\{R\left|R\;is\;optimistically\;D-achievable\;with\;the\;given\;f-divergence\right.\right\}. \end{array}$$

We similarly define the second-order achievability in the optimistic scenario.

**Definition 13.**  *L is said to be optimistically (D,R)-achievable with the given f-divergence if there exists a sequence of mapping $\phi_{n}:U_{M_{n}}\to X^{n}$ satisfying*

(197) $$\begin{array}{c} \underset{n\to \infty }{lim sup}D_{f}(X^{n}\left|\right|\phi_{n}\left(U_{M_{n}}\right))\leq D,\;\underset{n\to \infty }{lim inf}\frac{1}{\sqrt{n}}\log\frac{M_{n}}{e^{nR}}\leq L \end{array}$$

**Definition 14** (Optimistic second-order optimum resolvability rate).
(198) $$\begin{array}{c} T_{r}^{\left(f\right)}\left(D,R|X\right):=\inf\left\{L\left|L\;is\;optimistically\;(D,R)-achievable\;withth\;egiven\;f-divergence\right.\right\}. \end{array}$$

**Remark 12.**  *Conditions of optimistic D-achievability (195) can also be written as*

(199) $$\underset{n\to \infty }{lim inf}D_{f}(X^{n}\left|\right|\phi_{n}\left(U_{M_{n}}\right))\leq D,\;\underset{n\to \infty }{lim sup}\frac{1}{n}\log M_{n}\leq R.$$

*In actuality, the optimistic first-order optimum resolvability rate on the basis of (199) coincides with the one defined by Definition 12. A similar argument is also applicable in the optimistic second-order optimum resolvability rate as well as the optimistic optimum intrinsic randomness rates.*

The following theorem can be obtained by using Theorems 3 and 4.

**Theorem 13.**  *Under conditions C2’) and C3), for any 0≤D<f(0), it holds that*

(200) $$\begin{array}{cc} T_{r}^{\left(f\right)}\left(D|X\right) & =\lim_{\nu ↓0}\underset{n\to \infty }{lim inf}\frac{1}{n}H_{0}\left(1-f_{0}^{-1}\left(D+\nu \right)|X^{n}\right), \end{array}$$

*where $f_{0}$ is the offset function of f, defined in (131).*

**Proof.**  The proof proceeds in parallel with proof of Theorems 5 and 9 in which

${lim sup}_{n\to \infty }1/n\log M_{n}$ is replaced by ${lim inf}_{n\to \infty }1/n\log M_{n}$. □

**Theorem 14.**  *Under conditions C2’) and C3’), for any 0≤D<f(0), it holds that*

(201) $$\begin{array}{cc} T_{r}^{\left(f\right)}\left(D,R|X\right) & =\lim_{\nu ↓0}\underset{n\to \infty }{lim inf}\frac{H_{0}\left(1-f_{0}^{-1}\left(D+\nu \right)|X^{n}\right)-nR}{\sqrt{n}}. \end{array}$$

**Proof.**  The proof proceeds in parallel with the proof of Theorem 11 in which

${lim sup}_{n\to \infty }1/\sqrt{n}\log M_{n}$ is replaced by ${lim inf}_{n\to \infty }1/\sqrt{n}\log M_{n}$. □

We have revealed the first- and second-order optimum resolvability rates in the optimistic scenario. As a result, the effectiveness of Theorems 3 and 4 has also been shown.

The optimistic second-order optimum achievable rates with the half variational distance using the smooth Rényi entropy have already been derived by Tagashira and Uyematsu [31]. If we consider the case of $f\left(t\right)={\left(1-t\right)}^{+}$, Theorem 14 coincides with their result.

### 8.2. Intrinsic Randomness

We next consider the optimum intrinsic randomness rates in the optimistic scenario.

**Definition 15.**  *R is said to be optimistically Δ-achievable with the given f-divergence if there exists a sequence of mapping $\varphi_{n}:X^{n}\to U_{M_{n}}$ satisfying*

(202) $$\begin{array}{cc} \underset{n\to \infty }{lim sup}D_{f}(\varphi_{n}\left(X^{n}\right)\left|\right|U_{M_{n}}) & \leq \Delta ,\;\underset{n\to \infty }{lim sup}\frac{1}{n}\log M_{n}\geq R. \end{array}$$

**Definition 16** (Optimistic first-order optimum intrinsic randomness rate).
(203) $$T_{\iota }^{\left(f\right)}\left(\Delta |X\right):=\sup\left\{R\left|R\;is\;\Delta -achievable\;with\;the\;given\;f-divergence\right.\right\}.$$

**Definition 17.**  *L is said to be optimistically (Δ,R)-achievable with the given f-divergence if there exists a sequence of mapping $\varphi_{n}:X^{n}\to U_{M_{n}}$ satisfying*

(204) $$\begin{array}{cc} \underset{n\to \infty }{lim sup}D_{f}(\varphi_{n}\left(X^{n}\right)\left|\right|U_{M_{n}}) & \leq \Delta ,\;\underset{n\to \infty }{lim sup}\frac{1}{\sqrt{n}}\log\frac{M_{n}}{e^{nR}}\geq L. \end{array}$$

**Definition 18** (Optimistic second-order optimum intrinsic randomness rate).
(205) $$T_{\iota }^{\left(f\right)}\left(\Delta ,R|X\right):=\sup\left\{L\left|L\;is\;optimistically\;(\Delta ,R)-achievable\;with\;the\;givenf-divergence\right.\right\}.$$

Then, we have the following theorem by using Theorems 6 and 7.

**Theorem 15.**  *Under condition C2’), for any 0≤Δ<f(0) it holds that*

(206) $$\begin{array}{cc} T_{\iota }^{\left(f\right)}\left(\Delta |X\right) & =\lim_{\nu ↓0}\underset{n\to \infty }{lim sup}\frac{1}{n}H_{\infty }\left(1-f_{0}^{-1}\left(\Delta +\nu \right)|X^{n}\right). \end{array}$$

**Proof.**  The proof is similar to the proof of Theorems 8 and 10 in which ${lim inf}_{n\to \infty }1/n\log M_{n}$ is replaced by ${lim sup}_{n\to \infty }1/n\log M_{n}$. □

**Theorem 16.**  *Under condition C2’), for any 0≤Δ<f(0) it holds that*

(207) $$\begin{array}{cc} T_{\iota }^{\left(f\right)}\left(\Delta ,R|X\right) & =\lim_{\nu ↓0}\underset{n\to \infty }{lim sup}\frac{H_{\infty }\left(1-f_{0}^{-1}\left(\Delta +\nu \right)|X^{n}\right)-nR}{\sqrt{n}}. \end{array}$$

**Proof.**  The proof is similar to the proof of Theorem 12 in which ${lim inf}_{n\to \infty }1/\sqrt{n}\log M_{n}$ is replaced by ${lim sup}_{n\to \infty }1/\sqrt{n}\log M_{n}$. □

We have revealed the first- and second-order optimum intrinsic randomness rates in an optimistic scenario. As in the case of the resolvability problem, the effectiveness of Theorems 6 and 7 has also been shown.

The optimistic first-order optimum intrinsic randomness rate with the half variational distance using the smooth Rényi entropy has been derived by Uyematsu and Kunimatsu [10], while the second-order one has been characterized by Namekawa and Uyematsu [32]. Our results (Theorems 15 and 16) are generalizations of their results.

It is important to acknowledge that the topic of optimistic optimum achievable rates has also been previously studied in [9]. Our analysis of $T_{\iota }\left(\Delta |X\right)$ and $T_{\iota }\left(\Delta ,R|X\right)$ relates to the optimistic optimum achievable rates for intrinsic randomness with variational distance, which were addressed in Theorems 2 and 3 of [9] using different information-theoretic quantities. It should be noted that his work encompasses the analysis of several optimal rates, including the optimistic optimum achievable rates.

## 9. Discussion

Theorems 5 and 8 (as well as Theorems 11 and 12) have shown a kind of *duality* of two optimum achievable rates in different random number generation problems in terms of the smooth Rényi entropy. It should be noted that in the case of the variational distance, Theorem 6 in [6] and Theorem 7 in [10] have implied the same *duality*.

As we have mentioned in Section 1, the optimum achievable rates $S_{r}^{\left(f\right)}\left(D|X\right)$ and $S_{\iota }^{\left(f\right)}\left(\Delta |X\right)$ have already been characterized by using the information spectrum quantity.

**Definition 19.** 
(208) $$\begin{array}{ccc} {\bar{K}}_{f}\left(\varepsilon |X\right) & := & \inf\left\{R\left|\underset{n\to \infty }{lim sup}f\left(\mathrm{Pr}\left\{\frac{1}{n}\log\frac{1}{P_{X^{n}}\left(X^{n}\right)}\leq R\right\}\right)\leq \varepsilon \right.\right\},\\ {\underline{K}}_{f}\left(\varepsilon |X\right) & := & \sup\left\{R\left|\underset{n\to \infty }{lim sup}f\left(\mathrm{Pr}\left\{\frac{1}{n}\log\frac{1}{P_{X^{n}}\left(X^{n}\right)}\geq R\right\}\right)\leq \varepsilon \right.\right\}. \end{array}$$

Then, using these two quantities the following theorem has already been given.

**Theorem 17**  (Nomura [4] ([Theorems 3.1 and 4.1])). *Under conditions C1)–C3), it holds that*

(209) $$\begin{array}{ccc} S_{r}^{\left(f\right)}\left(D|X\right) & = & {\bar{K}}_{f}\left(D|X\right), \end{array}$$

(210) $$\begin{array}{ccc} S_{\iota }^{\left(f\right)}\left(\Delta |X\right) & = & {\underline{K}}_{f}\left(\Delta |X\right). \end{array}$$

From the above theorem and Theorems 5 and 8, we obtain the following relationship.

**Theorem 18.**  *Under conditions C1)–C3), it holds that*

(211) $$\begin{array}{ccc} \lim_{\nu ↓0}\underset{n\to \infty }{lim sup}\frac{1}{n}H_{0}\left(1-f^{-1}\left(D+\nu \right)|X^{n}\right) & = & {\bar{K}}_{f}\left(D|X\right), \end{array}$$

(212) $$\begin{array}{ccc} \lim_{\nu ↓0}\underset{n\to \infty }{lim inf}\frac{1}{n}H_{\infty }\left(1-f^{-1}\left(\Delta +\nu \right)|X^{n}\right) & = & {\underline{K}}_{f}\left(\Delta |X\right). \end{array}$$

The above theorem shows equivalences between information spectrum quantities and smooth Rényi entropies.

**Remark 13.**  *Theorem 18 can also be proved by using previous results and the continuity of the function f. In actuality, for $f\left(t\right)={\left(1-t\right)}^{+}$, Steinberg and Verdú [2] have shown*

(213) $$S_{r}^{\left(f\right)}\left(D|X\right)=\inf\left\{R\left|\underset{n\to \infty }{lim sup}\mathrm{Pr}\left\{\frac{1}{n}\log\frac{1}{P_{X^{n}}\left(X^{n}\right)}\geq R\right\}\leq D\right.\right\},$$

*from which together with the theorem given by Uyematsu [6] ([Theorem 6]) (Corollary 1 in this paper), we obtain*

(214) $$\lim_{\nu ↓0}\underset{n\to \infty }{lim sup}\frac{1}{n}H_{0}\left(D+\nu |X^{n}\right)=\inf\left\{R\left|\underset{n\to \infty }{lim sup}\mathrm{Pr}\left\{\frac{1}{n}\log\frac{1}{P_{X^{n}}\left(X^{n}\right)}\geq R\right\}\leq D\right.\right\}.$$

(215) $$\begin{array}{c} {\bar{K}}_{f}\left(D|X\right)=\inf\left\{R\left|\underset{n\to \infty }{lim sup}\mathrm{Pr}\left\{\frac{1}{n}\log\frac{1}{P_{X^{n}}\left(X^{n}\right)}\geq R\right\}\leq 1-f^{-1}\left(D\right)\right.\right\} \end{array}$$

*holds under conditions C1)–C3), we have (211). Equation (212) can also be derived from Corollary 1 and the result given by [8] ([Theorem 2.4.2]).*

**Remark 14.**  *From Definition 19, two quantities ${\bar{K}}_{f}\left(D|X\right)$ and ${\underline{K}}_{f}\left(D|X\right)$ are right-continuous functions of D, while*

(216) $$\underset{n\to \infty }{lim sup}\frac{1}{n}H_{0}\left(1-f^{-1}\left(D\right)|X^{n}\right)and\underset{n\to \infty }{lim inf}\frac{1}{n}H_{\infty }\left(1-f^{-1}\left(D\right)|X^{n}\right)$$

*may not. The operation $\lim_{\nu ↓0}$ in Theorem 18 can be considered an operation that makes quantities in (216) to be right-continuous. Furthermore, since $f^{-1}\left(D\right)$ is a decreasing function of D, $H_{\alpha }\left(1-f^{-1}\left(D\right)|X^{n}\right)$ is also a decreasing function of D. This means that the relation*

(217) $$\underset{n\to \infty }{lim sup}\frac{1}{n}H_{0}\left(1-f^{-1}\left(D\right)|X^{n}\right)\geq \lim_{\nu ↓0}\underset{n\to \infty }{lim sup}\frac{1}{n}H_{0}\left(1-f^{-1}\left(D+\nu \right)|X^{n}\right),$$

*holds. It should be emphasized that the above inequality holds with equality except for at most countably many D. Similarly, we obtain*

(218) $$\underset{n\to \infty }{lim inf}\frac{1}{n}H_{\infty }\left(1-f^{-1}\left(\Delta \right)|X^{n}\right)\geq \lim_{\nu ↓0}\underset{n\to \infty }{lim inf}\frac{1}{n}H_{\infty }\left(1-f^{-1}\left(\Delta +\nu \right)|X^{n}\right),$$

*where the equality holds except for at most countably many Δ. A similar observation can be applied to Theorem 20 below.*

The quantity on the right-hand side of Equation (214) is an information-spectrum quantity defined in [8]. This quantity has been instrumental in analyzing various problems, including source coding and resolvability. On the other hand, the following quantity is specifically used for analyzing the intrinsic randomness problem:

(219) $$\sup\left\{R\left|\underset{n\to \infty }{lim sup}\mathrm{Pr}\left\{\frac{1}{n}\log\frac{1}{P_{X^{n}}\left(X^{n}\right)}\leq R\right\}\leq D\right.\right\}$$

It is noteworthy that Hayashi [9] has defined second-order extensions of these quantities, further expanding their applicability in information theory. These extensions provide a more refined analysis of the asymptotic behavior of various information-theoretic problems.

We next consider the case of the *second-order* setting. We first define two quantities:

(220) $$\begin{array}{ccc} {\bar{K}}_{f}\left(\varepsilon ,R|X\right) & := & \inf\left\{L\left|\underset{n\to \infty }{lim sup}f\left(\mathrm{Pr}\left\{\frac{1}{n}\log\frac{1}{P_{X^{n}}\left(X^{n}\right)}\leq R+\frac{L}{\sqrt{n}}\right\}\right)\leq \varepsilon \right.\right\}, \end{array}$$

(221) $$\begin{array}{ccc} {\underline{K}}_{f}\left(\varepsilon ,R|X\right) & := & \sup\left\{L\left|\underset{n\to \infty }{lim sup}f\left(\mathrm{Pr}\left\{\frac{1}{n}\log\frac{1}{P_{X^{n}}\left(X^{n}\right)}\geq R+\frac{L}{\sqrt{n}}\right\}\right)\leq \varepsilon \right.\right\}. \end{array}$$

By using these quantities, the following theorem has been obtained.

**Theorem 19**  (Nomura [4] ([Theorems 6.1 and 6.2])). *Under conditions C1), C2) and C3’), it holds that*

(222) $$\begin{array}{ccc} S_{r}^{\left(f\right)}\left(D,R|X\right) & = & {\bar{K}}_{f}\left(D,R|X\right), \end{array}$$

(223) $$\begin{array}{ccc} S_{\iota }^{\left(f\right)}\left(\Delta ,R|X\right) & = & {\underline{K}}_{f}\left(\Delta ,R|X\right). \end{array}$$

From the above theorem and Theorems 11 and 12, we obtain:

**Theorem 20.**  *Under conditions C1), C2) and C3’), it holds that*

(224) $$\begin{array}{ccc} \lim_{\nu ↓0}\underset{n\to \infty }{lim sup}\frac{H_{0}\left(1-f^{-1}\left(D+\nu \right)|X^{n}\right)-nR}{\sqrt{n}} & = & {\bar{K}}_{f}\left(D,R|X\right), \end{array}$$

(225) $$\begin{array}{ccc} \lim_{\nu ↓0}\underset{n\to \infty }{lim inf}\frac{H_{\infty }\left(1-f^{-1}\left(\Delta +\nu \right)|X^{n}\right)-nR}{\sqrt{n}} & = & {\underline{K}}_{f}\left(\Delta ,R|X\right). \end{array}$$

The above theorem also shows equivalences between information spectrum quantities and smooth Rényi entropies in the *second-order* sense.

We have discussed functions *f* under C1), C2), and C3) (or C3’)) for simplicity. We can also extend the discussions for *f* under C2’) and C3) (or C3’)) with due modification using $f_{0}$.

## 10. Concluding Remarks

We have so far considered the optimum achievable rates in two random number generation problems with respect to a subclass of *f*-divergences. We have demonstrated *general formulas* of the *first-* and *second-order* optimum achievable rates with respect to the given *f*-divergence by using the smooth Rényi entropy including the inverse function of *f*. To our knowledge, this is the first use of the smooth Rényi entropy in information theory that contains the general function *f*. We believe that this is important from both the theoretical and practical viewpoints. In actuality, we have shown that we can easily derive the results on several important measures, such as the variational distance, the KL divergence, and the Hellinger distance, by substituting the specified function *f* into our *general formulas*. It should be noted that the optimum achievable rates with important measures have not been characterized before by using the smooth Rényi entropy except for the variational distance. Expressions of the smooth max entropy in Theorem 1 and the smooth min entropy in Theorem 2 are simple and easy to understand. Hence, our results using the smooth max entropy and the smooth min entropy are also comprehensive. This provides us another viewpoint to understand the mechanism of the random number generation problems compared to the results given in [4], in which the information spectrum quantities are used. In addition, we have shown that the conditions on *f*-divergence can be relaxed, leading to the general formulas holding for a wider class of *f*-divergence. These are major contributions of this paper.

As a consequence of our results and the results in [4], the equivalence of the smooth Rényi entropy and the information spectrum quantity has been clarified (Theorem 18). One may consider that if we show this equivalency first, then we can derive Theorems 5 and 8 directly. This observation is correct. That is, one simple method of deriving both of the general formulas of the optimum achievable rates (Theorems 5 and 8) is to show this equivalency (Theorem 18) first. Then, combining Theorem 18 and results in [4], we obtain Theorems 5 and 8. However, we have taken another approach to show Theorems 5 and 8 in this paper. For example, we first have shown Theorems 3 and 4 so as to establish Theorem 5. Although Theorem 5 has been established by using Theorems 3 and 4, we think that these two theorems are significant themselves. In fact, Theorem 3 provides us with how to construct an optimum mapping in the resolvability problem, and Theorem 4 shows the relationship between the rate of the random number and the smooth max entropy in terms of the finite block length. Hence, these two theorems are also significant not only for proving Theorem 5 but also for constructing the optimum mapping in the practical situation.

In this paper, we have considered the *f*-divergence $D_{f}(X^{n}\left|\right|\phi_{n}\left(U_{M_{n}}\right))$ in the case of the resolvability problem and $D_{f}(\varphi_{n}\left(X^{n}\right)\left|\right|U_{M_{n}})$ in the case of the intrinsic randomness problem and shown a kind of *duality* of these problems in terms of the smooth Rényi entropy. On the other hand, we can consider the resolvability problem with respect to $D_{f}(\phi_{n}\left(U_{M_{n}}\right)\left|\right|X^{n})$ as well as the intrinsic randomness problem with respect to $D_{f}(U_{M_{n}}\left|\right|\varphi_{n}\left(X^{n}\right))$. Although these problems are also important, a similar technique in the present paper cannot be applied directly. In order to treat these problems, it seems we need some novel techniques, which remain to be studied. This is similar to the case of the information spectrum approach [4].

Finally, the condition C3) and the assumption (15) for the source, have only been needed to show Direct Part (Theorem 3) in the resolvability problem. To consider the necessity or weakening of these conditions is also a future work.

## Data Availability

No new data were created or analyzed in this study. Data sharing is not applicable to this article.
